# Supplementary material for: The Singularity of Cetacea Behavior Parallels the Complete Inactivation of Melatonin Gene Modules
Source: Genes (Basel). 2019 Feb 6;10(2):121. doi: 10.3390/genes10020121 (PMC6410235; doi:10.3390/genes10020121)
Supplement: Supplementary file 1 [file genes-10-00121-s001.pdf]

<u>Supplementary information</u>

Full Title: The singularity of Cetacea behavior parallels the complete inactivation of melatonin gene modules

Short Title: Melatonin gene modules are eroded in Cetacea

Mónica Lopes-Marques[1•], Raquel Ruivo[1•*], Luís Q. Alves[1,2], Nelson Sousa[1], André M. Machado[1], L. Filipe C. Castro[1,2*]

[1]CIIMAR/CIMAR - Interdisciplinary Centre of Marine and Environmental Research, University of Porto, Portugal

[2]FCUP - Faculty of Sciences, Department of Biology, University of Porto, Portugal

[•]Equal Contribution

*Correspondence: rruivo@ciimar.up.pt; filipe.castro@ciimar.up.pt

**Figure S1:** Comparative synteny maps of *Aanat* genomic locus.	3
**Figure S2:** Multiple alignment of the predicted exon 1 of *Aanat* in Cetacea.	4
**Figure S3:** SRA validation for conserved stop mutation in exon 1 of *Aanat* in Cetacea.	5
**Figure S4:** Comparative synteny maps of *Asmt* genomic *locus*.	10
**Figure S5:** Schematic representation of the Human ASMT isoform 1.	11
**Figure S6:** Multiple alignment of the predicted exon 1 of *Asmt* in Cetacea.	11
**Figure S7:** SRA validation for conserved frameshift mutation in exon 1 of *Asmt* in Cetacea.	12
**Figure S8:** Alignment of exon 5 of *Asmt* in multiple mammals.	15
**Figure S9:** SRA validation for frameshift mutation in exon 4 of *P. catodon Asmt*.	16
**Figure S10:** SRA validation for frameshift mutation in exon 3 of *L. obliquidens Asmt*.	16
**Figure S11:** Multiple alignment of the predicted *Mtnr1a* relic sequences (exon 1) in Cetacea.	17
**Figure S12:** SRA validation for 19 nucleotide deletion in exon 1 of *P. catodon Mtnr1a*.	17
**Figure S 3:** Comparative synteny maps of *Mtnr1b* genomic locus.	18
**Figure S14:** Multiple alignment of the predicted exon 1 of *Mtnr1b* in Cetacea.	19
**Figure S15:** SRA validation for stop mutation in exon 1 of *Mntr1b* from *O. orca*.	19
**Figure S16:** SRA validation for nucleotide insertion generating a premature stop mutation in exon 1 of the *Mntr1b* gene from *B. acutorostrata* and *E. robustus*.	20
**Figure S17:** SRA validation for loss of start codon in exon 1 of the *Mntr1b* gene from *L. obliquidens*.	21
**Figure S18:** Partial multiple alignment of the predicted exon 2 of *Mtnr1b* in Cetacea.	21
**Figure S19:** SRA validation for conserved nucleotide deletion in exon 2 of *Mtnr1b* in Cetacea.	22
**Figure S20:** SRA validation for nucleotide deletion in exon 2 of *Mtnr1b* from *E. robustus*.	25
**Figure S21:** *H. amphibius* exon 1 and 5 assembly.	26
**Figure S22:** Phylogenetic analysis of Perissodactyla, Artiodactyla and Primate *Aanat* genes.	27
**Figure S23:** Phylogenetic analysis of Perissodactyla, Artiodactyla and Primate *Mtnr1a* genes.	28
**Figure S24:** Phylogenetic analysis of Perissodactyla, Artiodactyla and Primate *Mtnr1b* genes.	29
**Figure S25:** *T. manatus latirostris* gene annotations.	30
**Figure S26:** SRA validation for loss of start codon in exon 1 of the *Aanat* gene from *T. manatus latirostris.*	31
**Figure S27:** SRA validation for premature stop codon in exon 5 of the *Asmt* gene from *T. manatus latirostris.*	31
**Figure S28:** SRA validation for single nucleotide insertion in exon 2 of the *Mtnr1a* gene from *T. manatus latirostris.*	32
**Figure S29:** SRA validation for frameshift mutation in exon 2 of the *Mtnr1b* gene from *T. manatus latirostris.*	32
**Table S1:** Blast-n output of *Bos Taurus* and *Homo sapiens* sequences against the *H. amphibius* genome.	33
**Table S2:** Accession numbers of the *Aanat* orthologues sequences used in phylogenetic analyses.	34
**Table S3:** Accession numbers of the *Mtnr1a* orthologues sequences used in phylogenetic analyses.	35
**Table S4:** Accession numbers of the *Mtnr1b* orthologues sequences used in phylogenetic analyses.	36
**Gene annotations:** In silico gene annotations of *Aanat*, *Mtnr1a* and *Mtnr1b* in *H. amphibius*	37

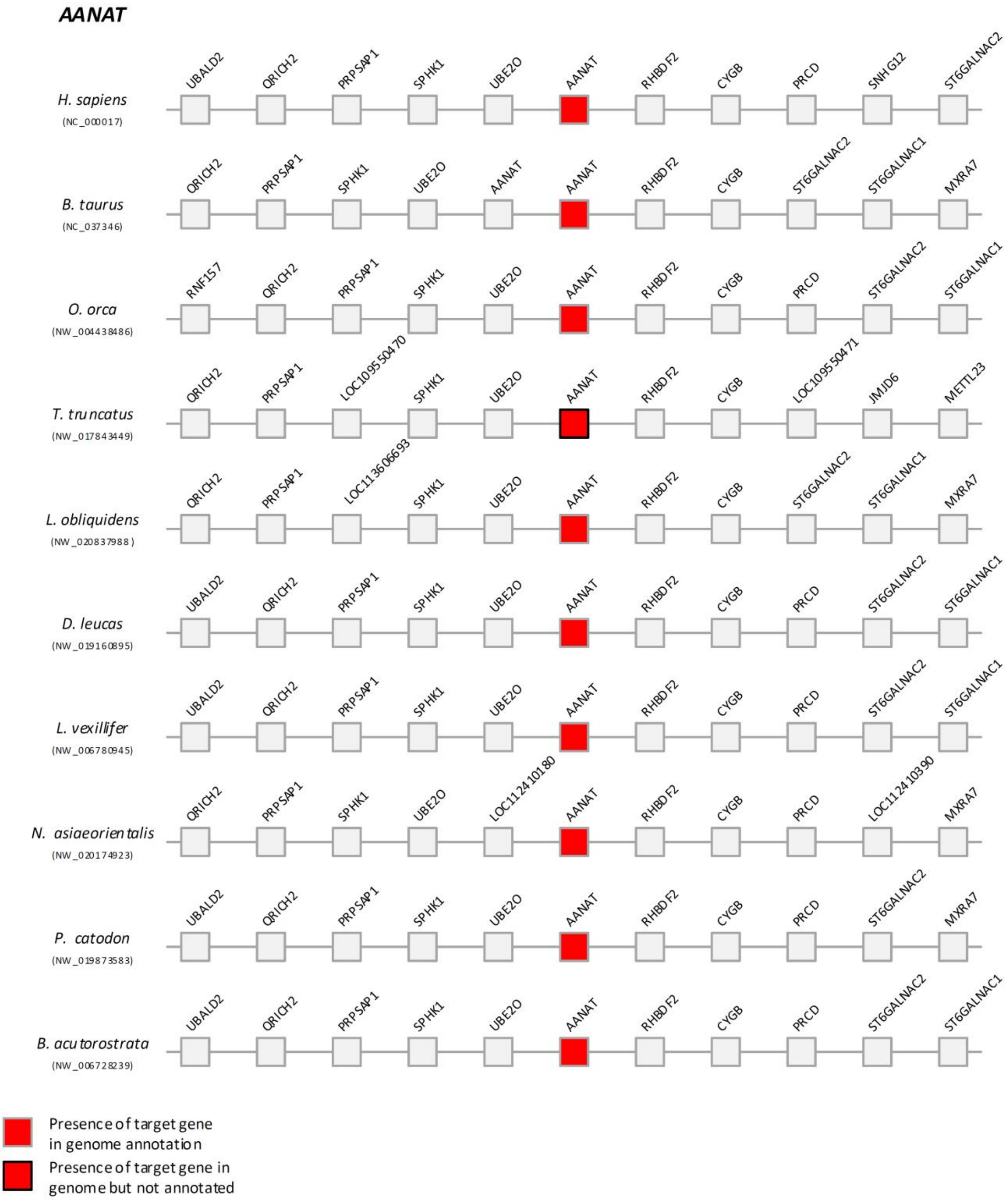

Figure S1: Comparative synteny maps of *Aanat* genomic *locus.*

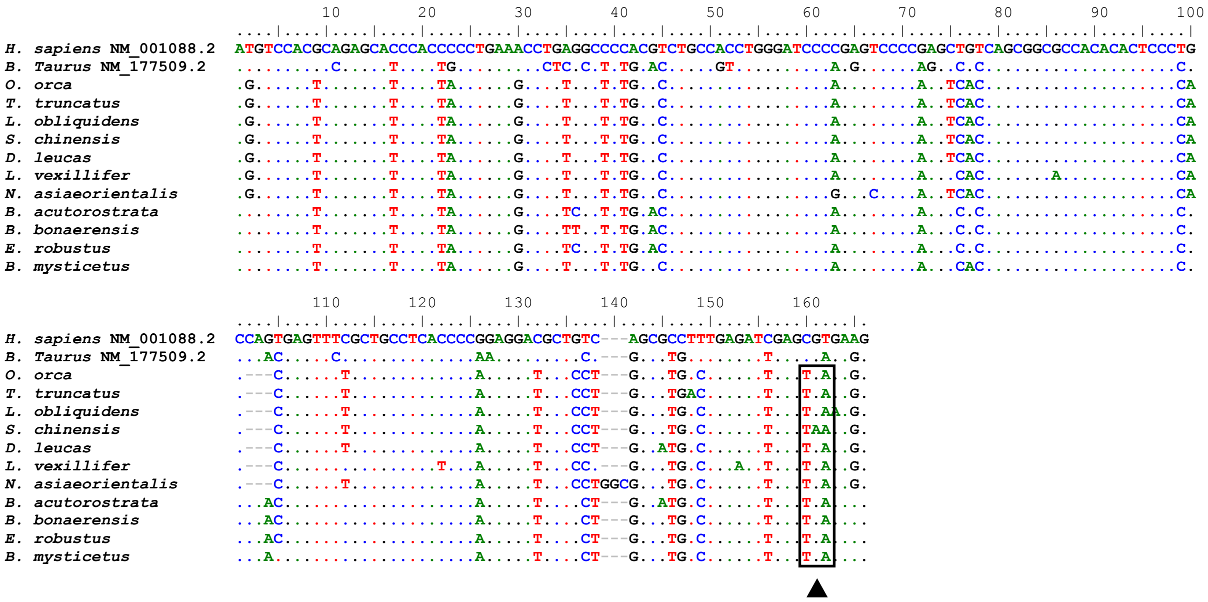

Figure S2: Multiple alignment of the predicted exon 1 of *Aanat* in the listed species. Conserved premature stop mutation, validated with SRA (when available), is represented in the corresponding position with a black arrow.

Orcinus orca
SRA serached
SRR1164379- University of Durham 2014-02-13 **Sample ID:** SAMN02595096 (AR-Genome)
SRR574978/81- Baylor College of Medicine 2012-09-20 **Sample ID:** SAMN01180276 (AForca1)

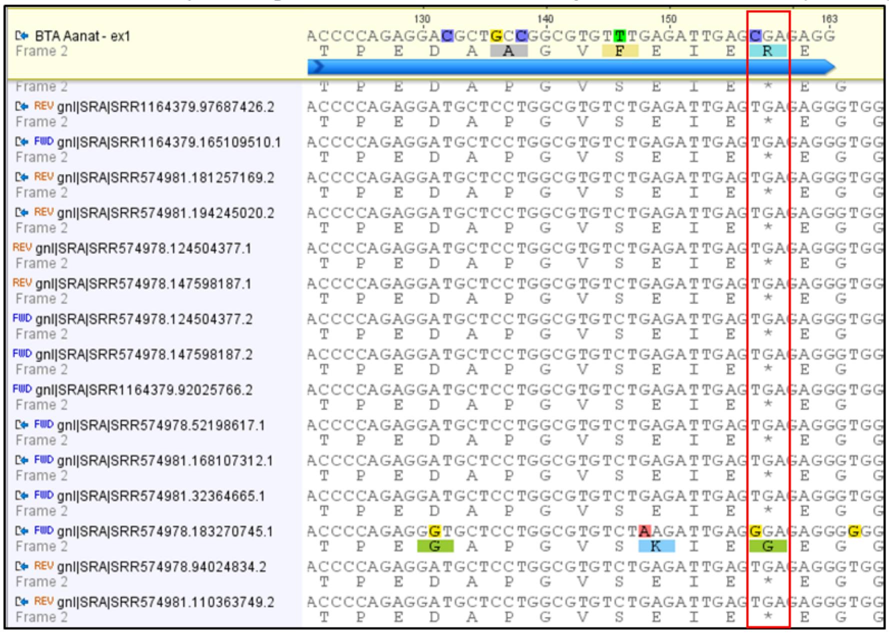

Tursiops truncatus
Trace Archive Blast
ti:1496398209
ti:1518196214

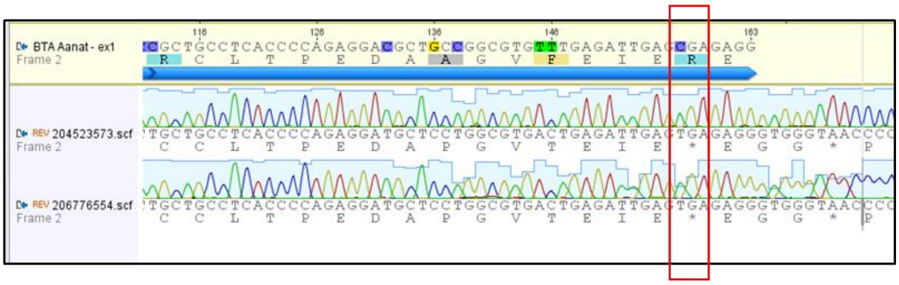

Tursiops truncatus

SRA searched

SRR606320/21/22/24- Baylor College of Medicine 2015-07-22 **Sample ID:** SAMN00000070

SRR2148843 - Beijing Genome Institute 2016-08-10 **Sample ID**:SAMN03968477

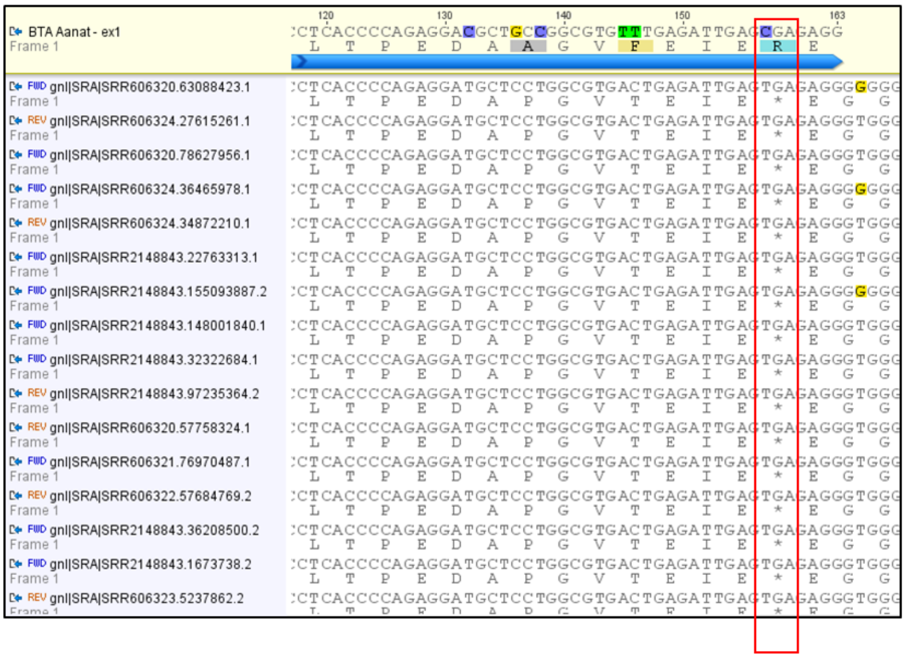

Lagenorhynchus obliquidens

SRA searched

SRR7349574 - BC Cancer Agency Michael Smith Genome Sciences Centre (BCCAGSC) 2018-06-21 **Sample ID:** SAMN09386610

SRR7345555 - BC Cancer Agency Michael Smith Genome Sciences Centre (BCCAGSC) 2018-06-21 **Sample ID:** SAMN09386610

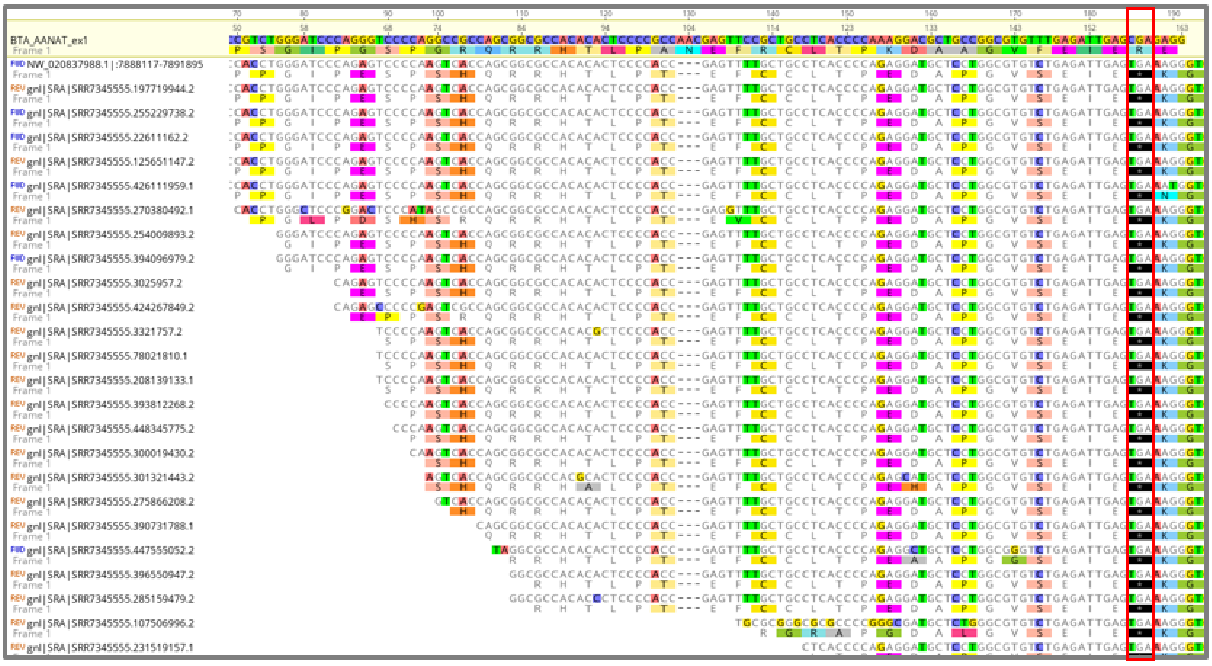

Delphinapterus leucas
SRA searched
SRR5197962 - BC Cancer Agency Michael Smith Genome Sciences Centre 2017-06-27 **Sample ID:** SAMN06217832 (Qila21)
SRR5659909 - BC Cancer Agency Michael Smith Genome Sciences Centre 2017-06-27 **Sample ID:** SAMN06216270 (Aurora29)

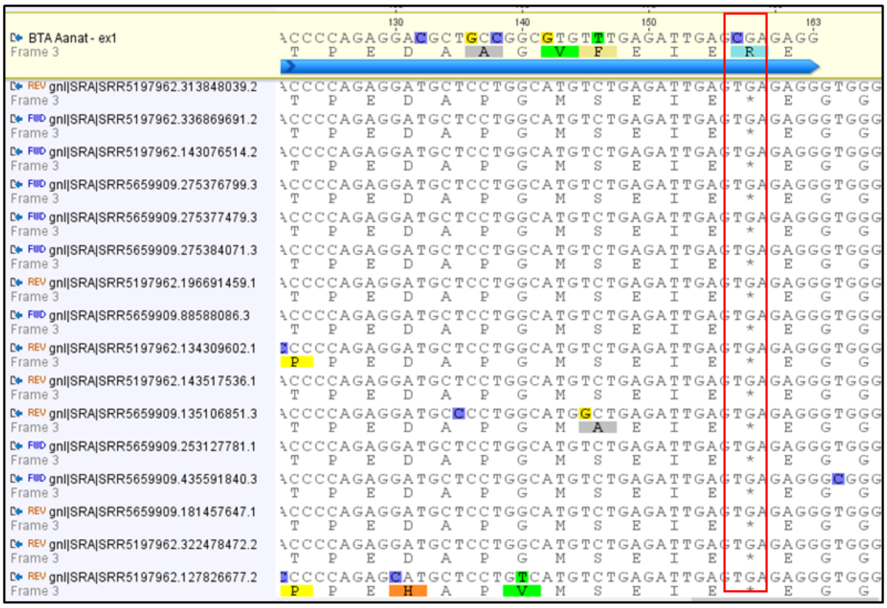

Neophocaena asiaeorientalis
SRA searched
SRR6923830 - Northwestern Polytechnical University 2018-04-08 **Sample ID:** SAMN08512128 (MCJT3)
SRR4292276- Beijing Genome Institute (BGI) 2017-09-23 **Sample ID:** SAMN05818630

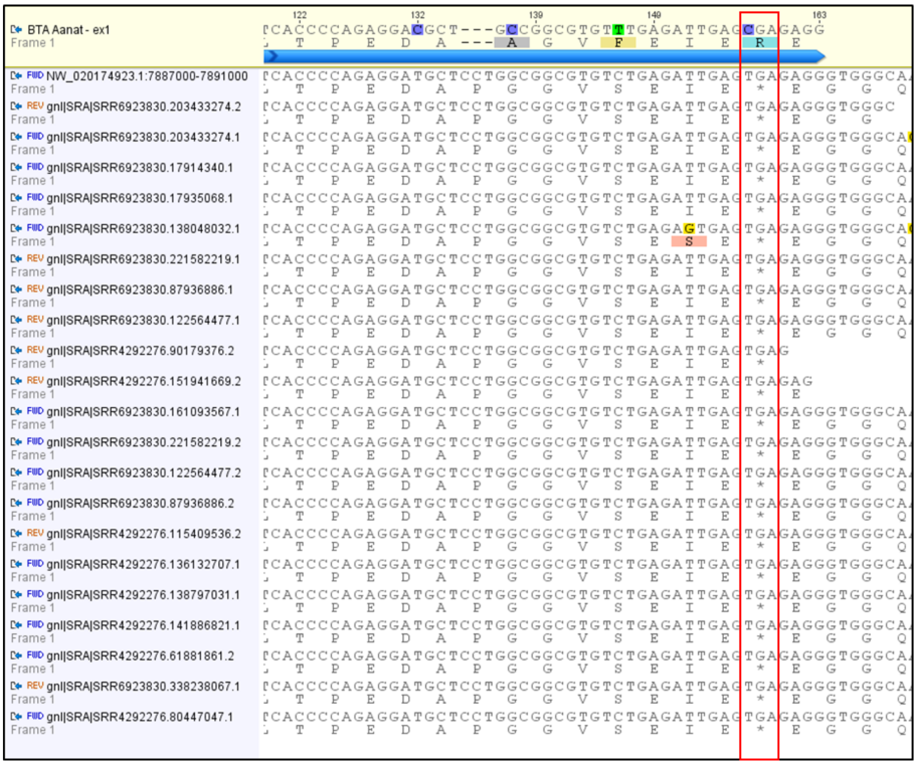

Balaenoptera acutorostrata

SRA searched

SRR924087- Korea Institute of Ocean Science and Technology 2013-10-31 **Sample ID:** SAMN02192644 (MinkeWhale-01)

SRR1802582 - Seoul National University 2016-02-11 **Sample ID:** SAMN03339797 (MW30)

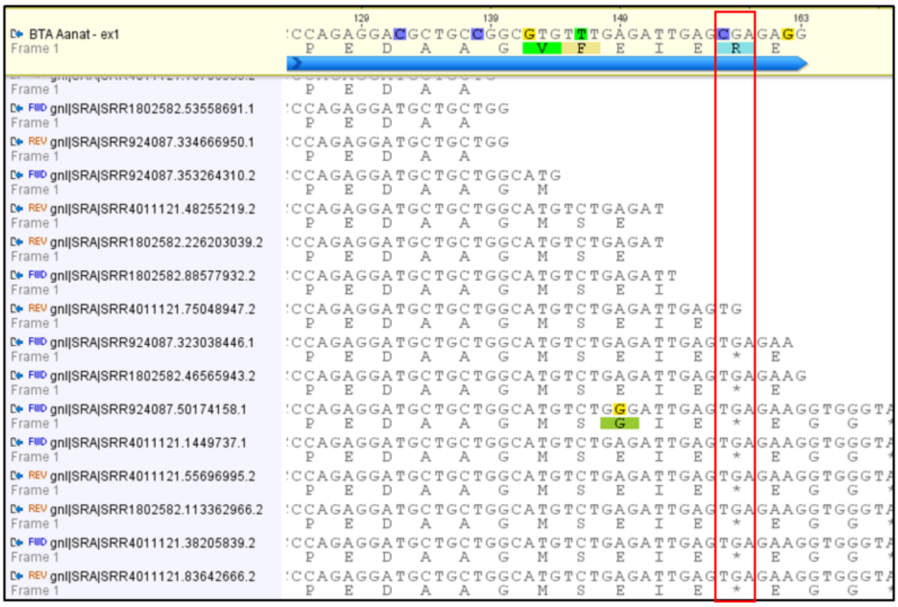

Balaenoptera bonaerensis

SRA searched

SRR4011113- Institute of Marine Research 2016-08-13 **Sample ID:** SAMN05447715

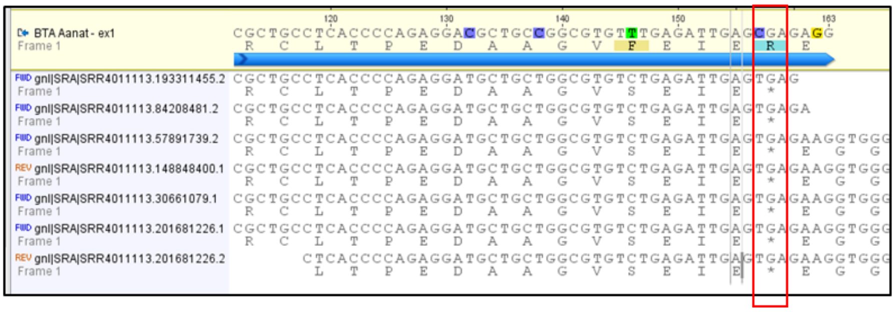

Eschrichtius robustus

SRA searched

SRR5495100- Purdue University 2017-05-02 **Sample ID:** SAMN06837694 (GFD-02 -female)

SRR5495108 - Purdue University 2017-05-02 **Sample ID:** SAMN06837692 (ER-14-168 -male)

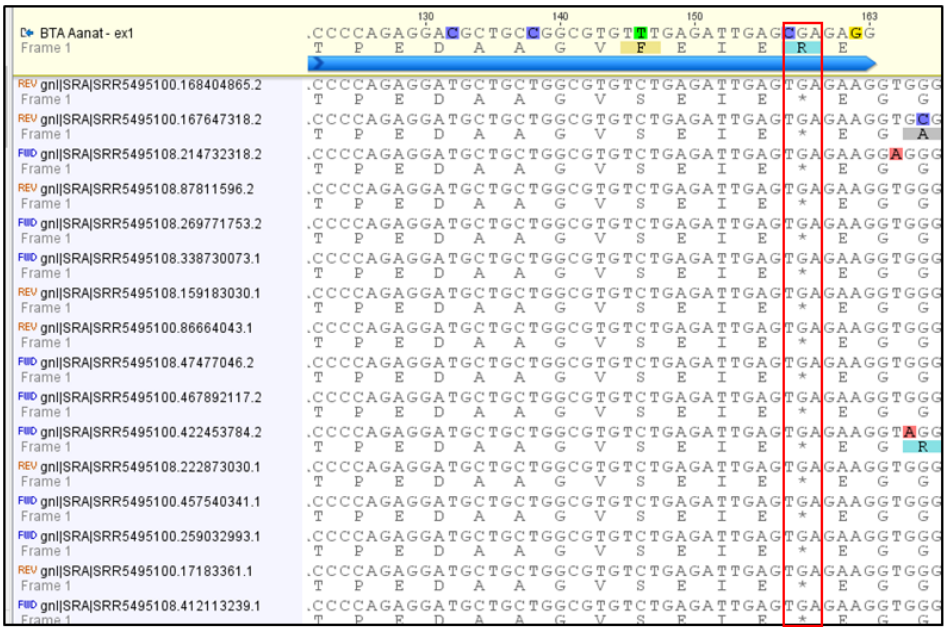

Balaena mysticetus

SRA searched

SRR1685383 - University of Liverpool 2014-12-31 **Sample ID:** SAMN03225705 (bowhead whale1)

SRR1685385 - University of Liverpool 2014-12-31 **Sample ID:** SAMN03225705 (bowhead whale1)

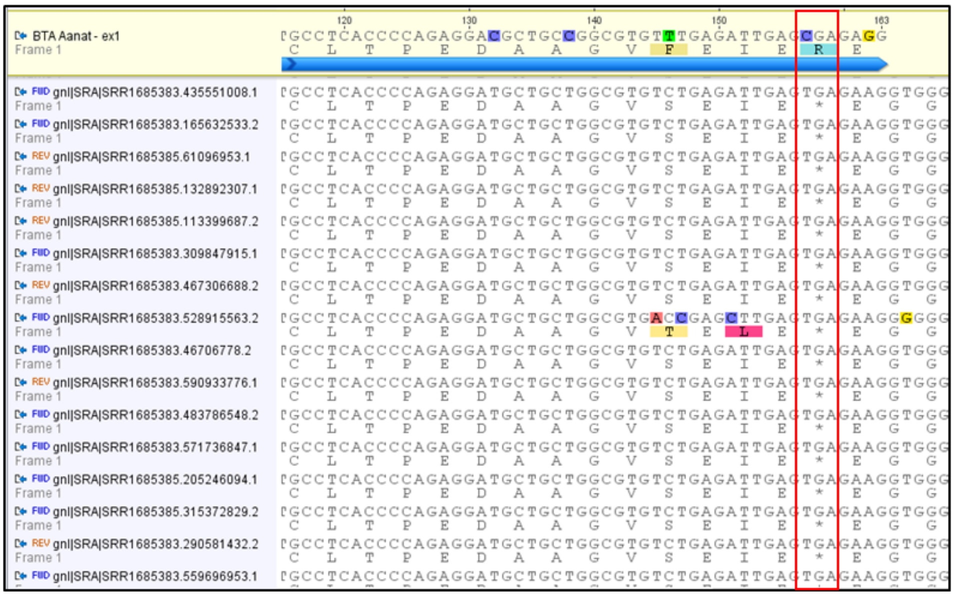

Figure S3: SRA validation for conserved stop mutation (red box) in exon 1 of the *Aanat* gene from *O. orca*, *T. truncatus*, *L. obliquidens*, *D. leucas*, *N. asiaeorientalis*, *B. acutorostrata*, *B. bonaerensis*, *E. robustus* and *B. mysticetus*.

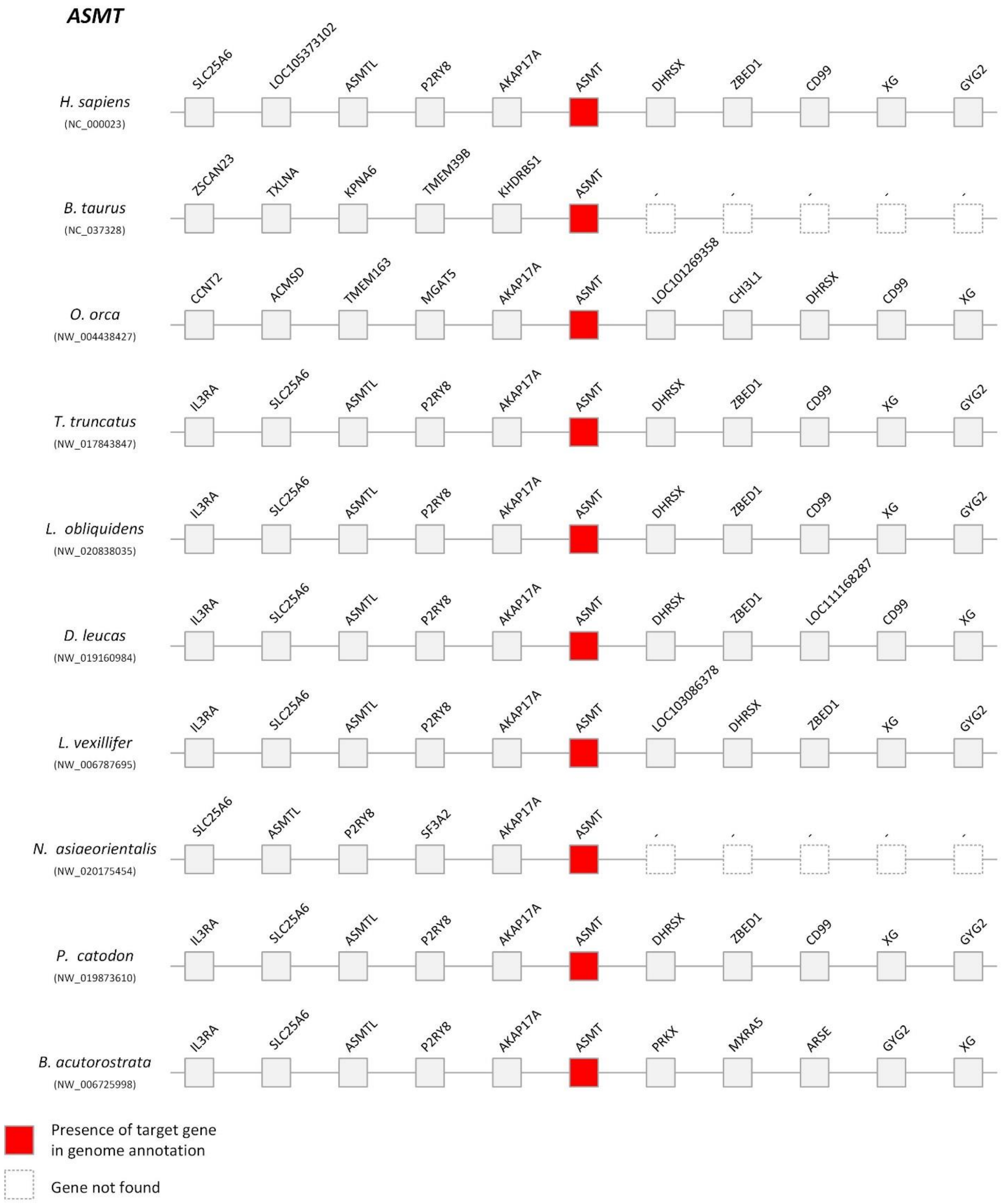

Figure S4: Comparative synteny maps of *Asmt* genomic *locus.*

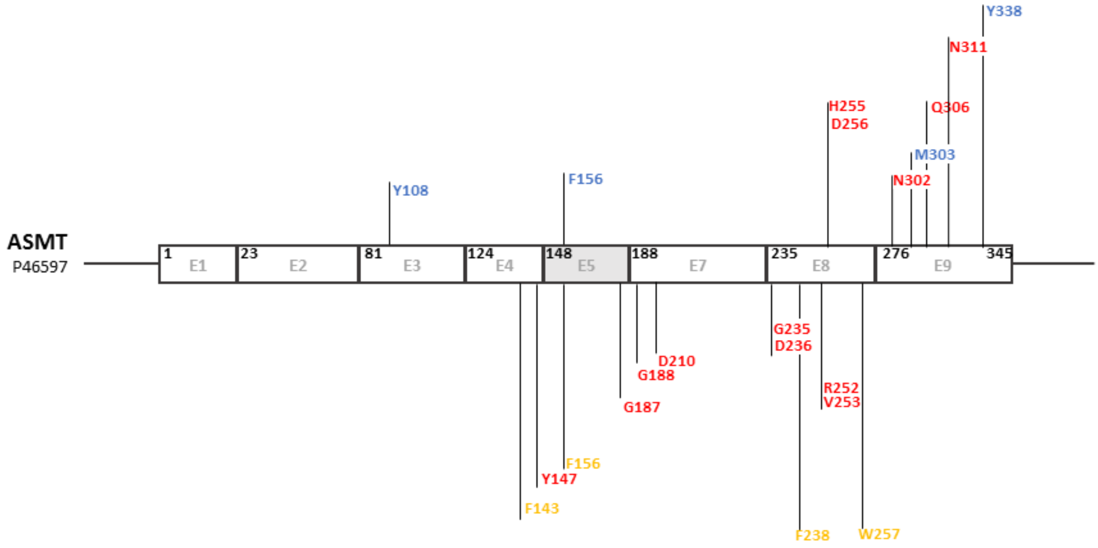

Figure S5: Schematic representation of the Human ASMT isoform 1 (not to scale). On top, in blue, relative location of conserved residues in proximity to the hydroxyl side of *N*-acetyl serotonin (NAS), in red residues that establish H-bonds to NAS. On the bottom in orange conserved aromatic residues that encircle the SAM binding site, in red residues that establish H-Bonds to SAM.

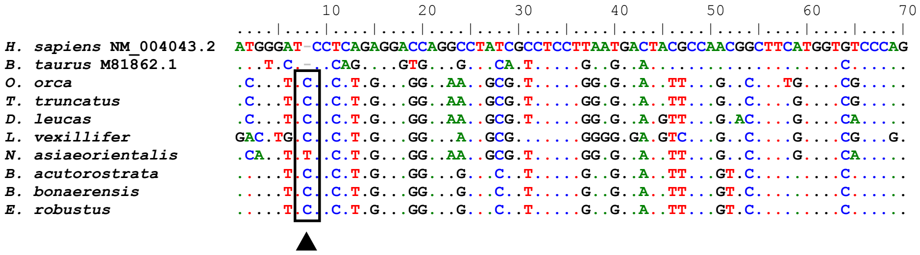

Figure S6: Multiple alignment of the predicted exon 1 of *Asmt* in the listed species. Conserved single nucleotide insertion, validated with SRA (when available), is represented in the corresponding position with a black arrow.

Orcinus orca

SRA serached

SRR1164379- University of Durham 2014-02-13 **Sample ID:** SAMN02595096 (AR-Genome)

SRR574977/82- Baylor College of Medicine 2012-09-20 **Sample ID:** SAMN01180276 (AForca1)

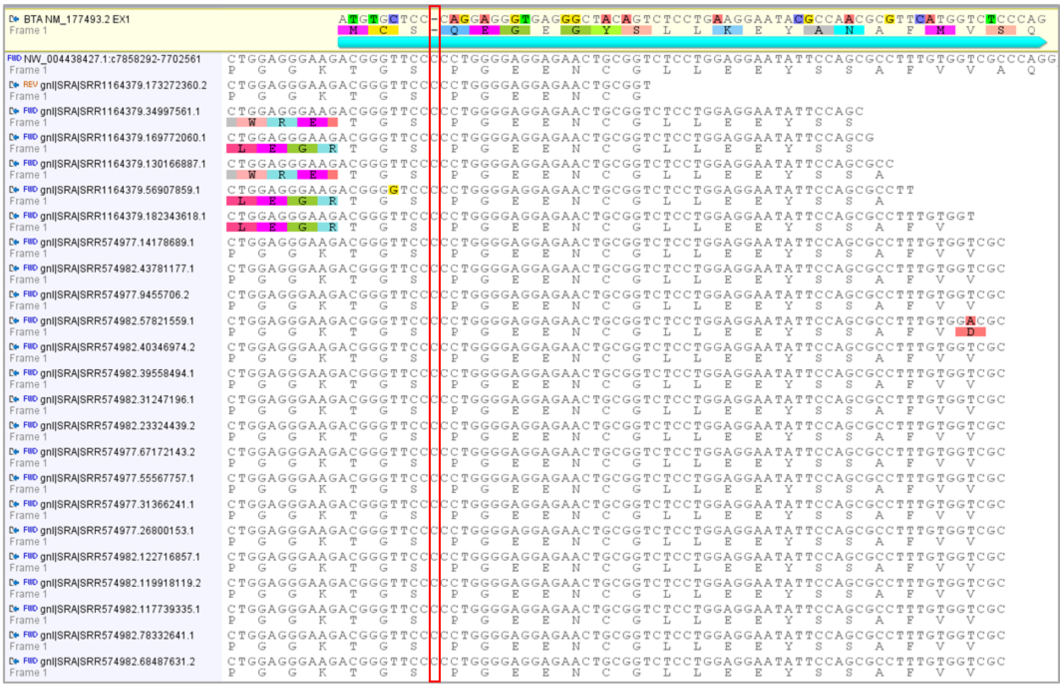

Tursiops truncatus

SRA searched

SRR5125024 - National Institute of Standards and Technology 2016-12-27 **Sample ID:**SAMN06114300

SRR606319/20/21/22/24- Baylor College of Medicine 2015-07-22 **Sample ID:** SAMN00000070

SRR2148845 - Beijing Genome Institute 2016-08-10 **Sample ID**:SAMN03968479

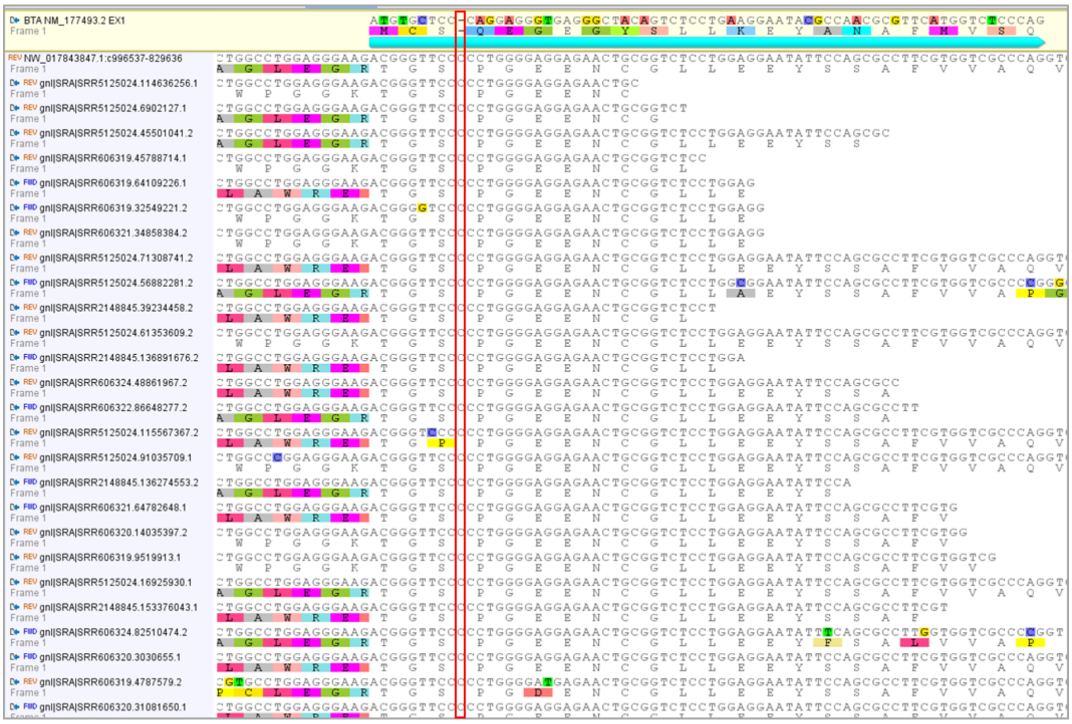

Tursiops truncatus
Trace Archive Blast
ti:1416172502

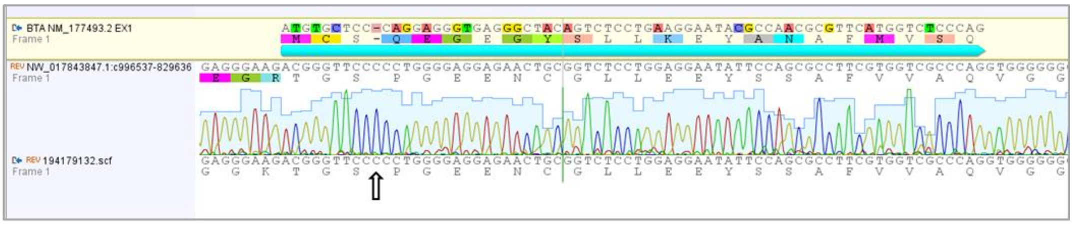

Delphinapterus leucas
SRA searched
SRR5197962 - BC Cancer Agency Michael Smith Genome Sciences Centre 2017-06-27 **Sample ID:** SAMN06217832 (Qila21)
SRR5197961 - BC Cancer Agency Michael Smith Genome Sciences Centre 2017-06-27 **Sample ID:** SAMN06216270 (Aurora29)

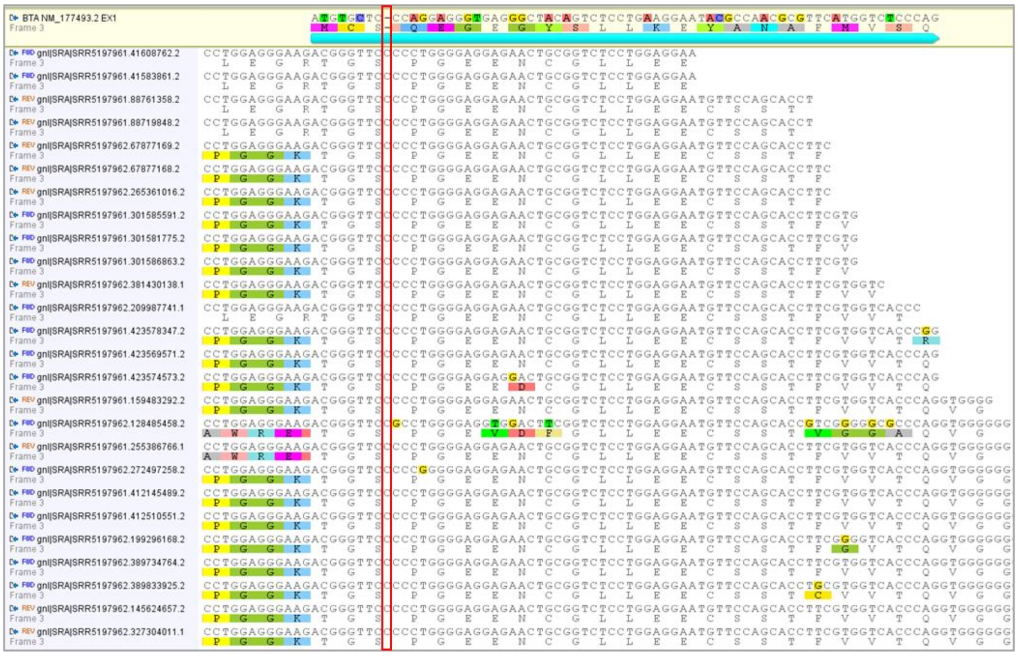

Neophocaena asiaeorientalis

SRA searched

SRR6923830 - Northwestern Polytechnical University 2018-04-08 **Sample ID:** SAMN08512128 (MCJT3)

SRR4292276- Beijing Genome Institute (BGI) 2017-09-23 **Sample ID:** SAMN05818630

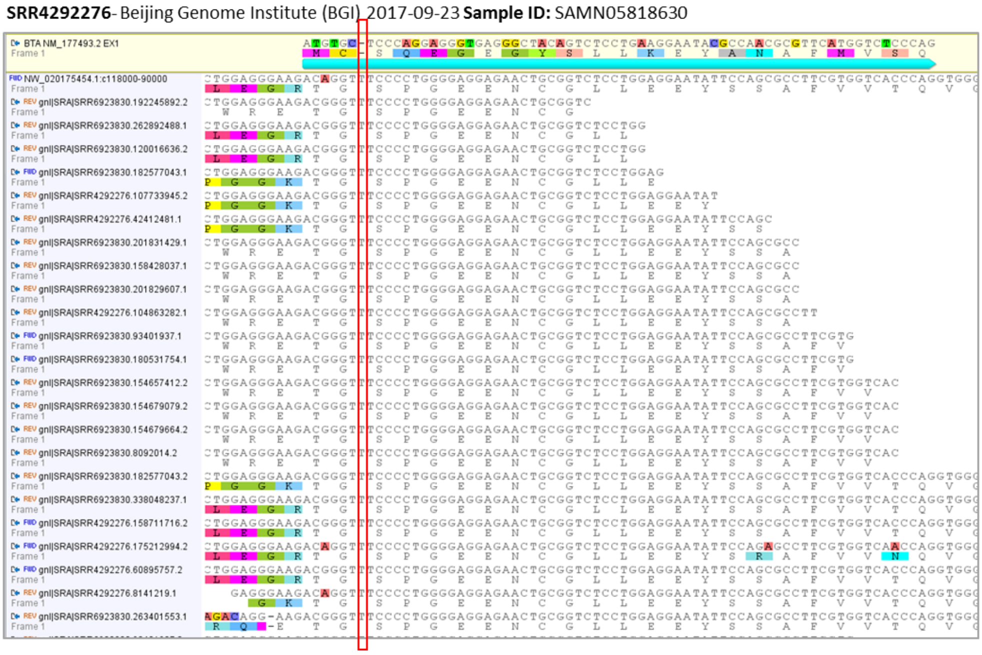

Balaenoptera acutorostrata

SRA searched

SRR924087- Korea Institute of Ocean Science and Technology 2013-10-31 **Sample ID:** SAMN02192644 (MinkeWhale-01)

SRR4011112 - Institute of Marine Research 2016-08-13 **Sample ID:** SAMN05447714 (AT)

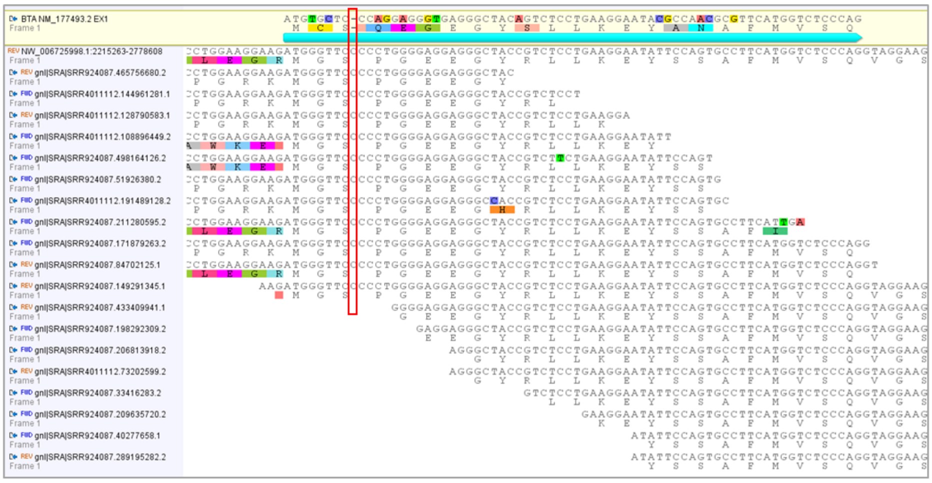

Eschrichtius robustus

SRA searched

SRR5495100- Purdue University 2017-05-02 **Sample ID:** SAMN06837694 (GFD-02 -female)

SRR5495106 - Purdue University 2017-05-02 **Sample ID:** SAMN06837692 (ER-14-168 -male)

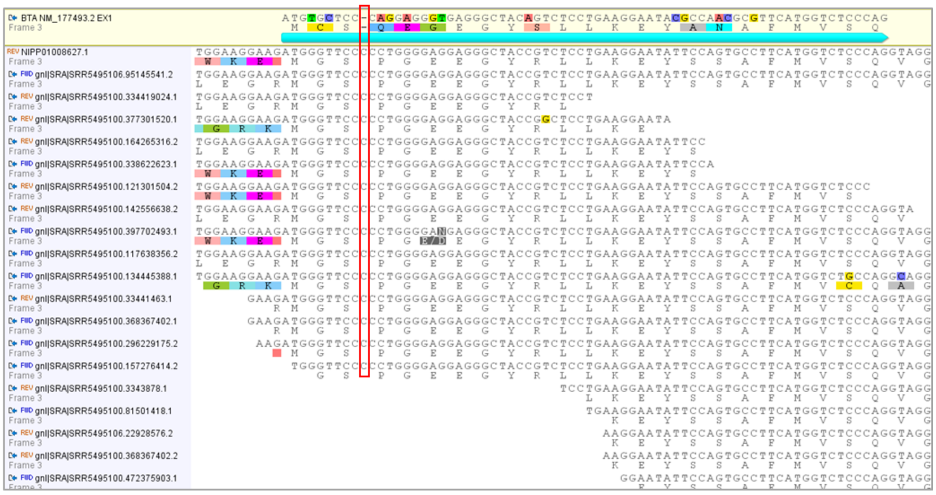

Figure S7: SRA validation for conserved frameshift mutation (red box) in exon 1 of the *Asmt* gene from *O. orca*, *T. truncatus*, *D. leucas*, *N. asiaeorientalis*, *B. acutorostrata* and *E. robustus*.

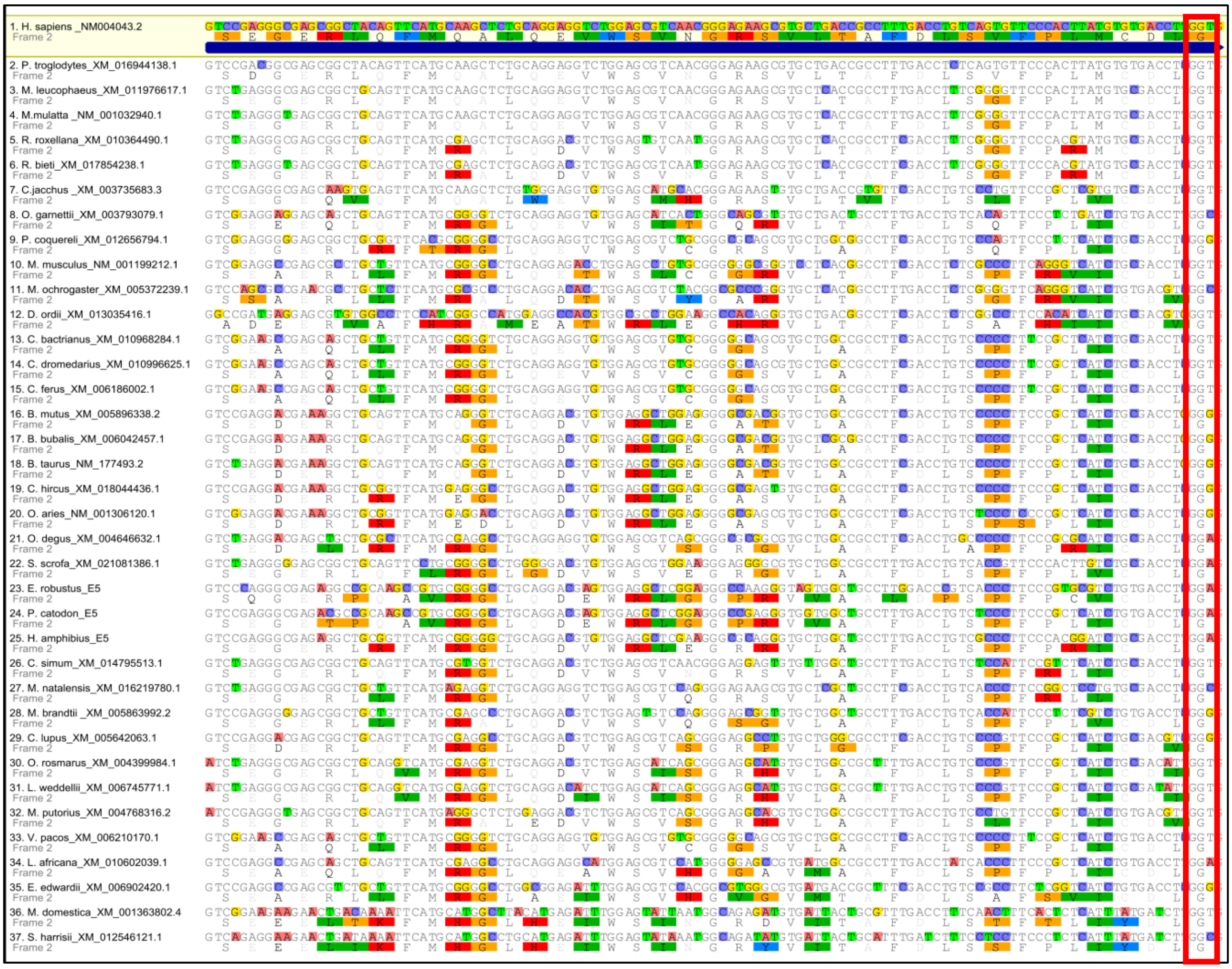

Figure S8: Alignment of exon 5 of *Asmt* in multiple mammals, red box indicates conserved Gly187 participating in the active site of ASMT.

Physeter catodon frameshift mutation 1 nt insertion in exon 4
SRA searched
SRR5146843- The Genome Center at Washington University School of Medicine in St. Louis 2017-01-05 **Sample ID:** SAMN06187413
SRR5146847- The Genome Center at Washington University School of Medicine in St. Louis 2017-01-05 **Sample ID:** SAMN06187412
SRR5146865- The Genome Center at Washington University School of Medicine in St. Louis 2017-01-05 **Sample ID:** SAMN06187411

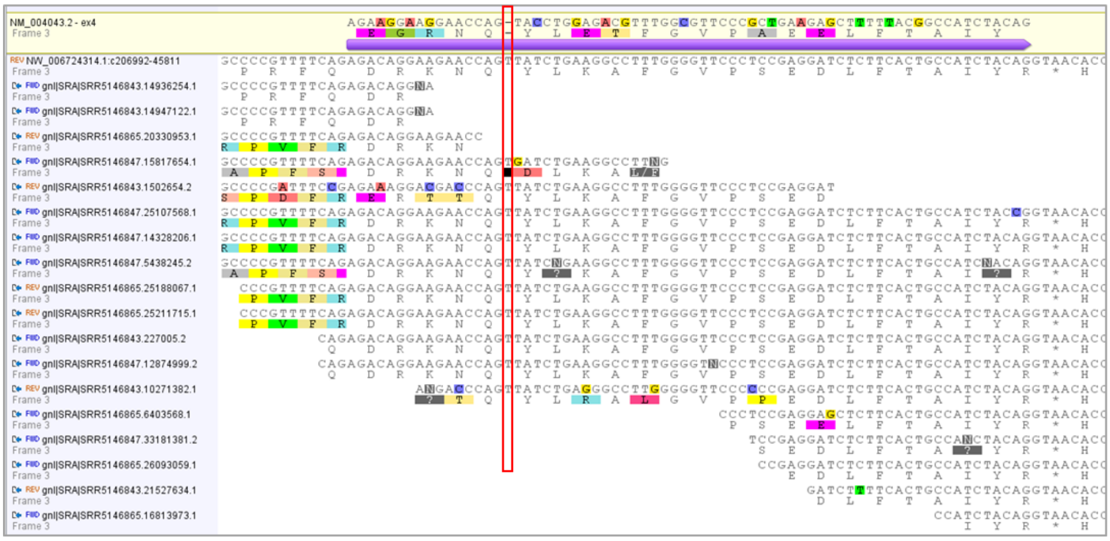

Figure S9: SRA validation for frameshift mutation (red box) in exon 4 of *P. catodon Asmt*.

Lagenorhynchus obliquidens 129
SRA searched
SRR7349574 - BC Cancer Agency Michael Smith Genome Sciences Centre (BCCAGSC) 2018-06-21 **Sample ID:** SAMN09386610 130
SRR7345555 - BC Cancer Agency Michael Smith Genome Sciences Centre (BCCAGSC) 2018-06-21 **Sample ID:** SAMN09386610

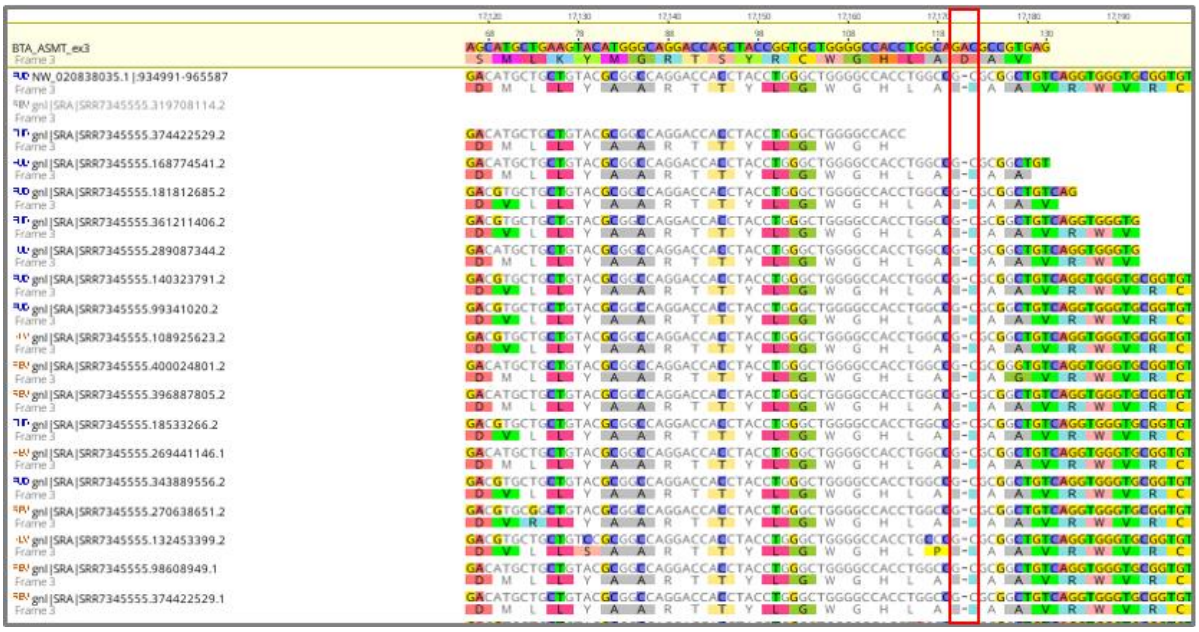

Figure S10: SRA validation for frameshift mutation (red box) in exon 3 of *L. obliquidens Asmt*.

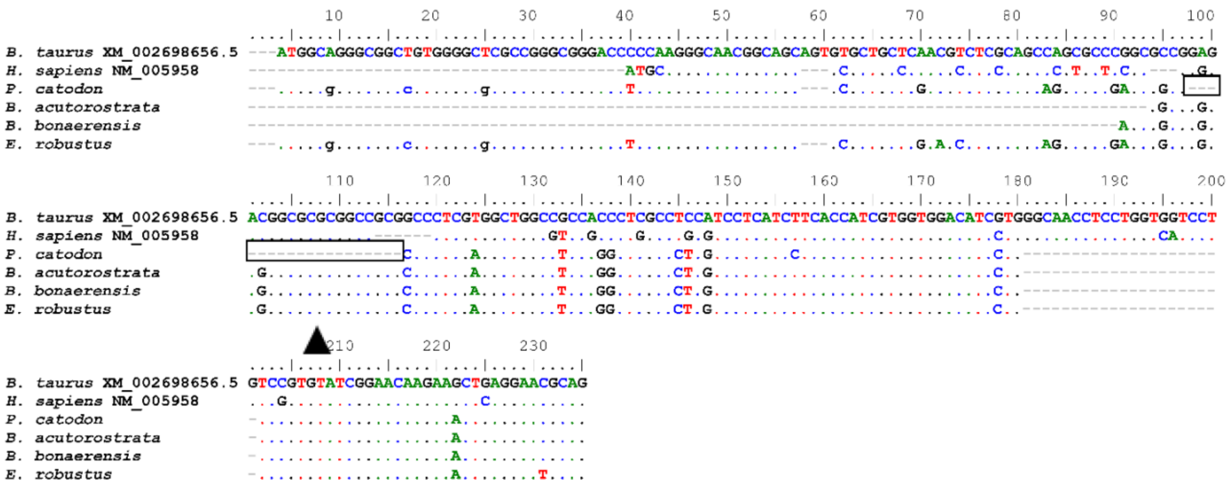

Figure S11: Multiple alignment of the predicted *Mtnr1a* relic sequences (exon 1) in the listed species. Nucleotide insertion of *P. catodon*, validated with SRA, is represented in the corresponding position with a black arrow

Physeter catodon frameshift mutation 19 nt deletion in exon 1
SRA searched
SRR5146843- The Genome Center at Washington University School of Medicine in St. Louis 2017-01-05 **Sample ID:** SAMN06187413
SRR5146847- The Genome Center at Washington University School of Medicine in St. Louis 2017-01-05 **Sample ID:** SAMN06187412
SRR5146865- The Genome Center at Washington University School of Medicine in St. Louis 2017-01-05 **Sample ID:** SAMN06187411

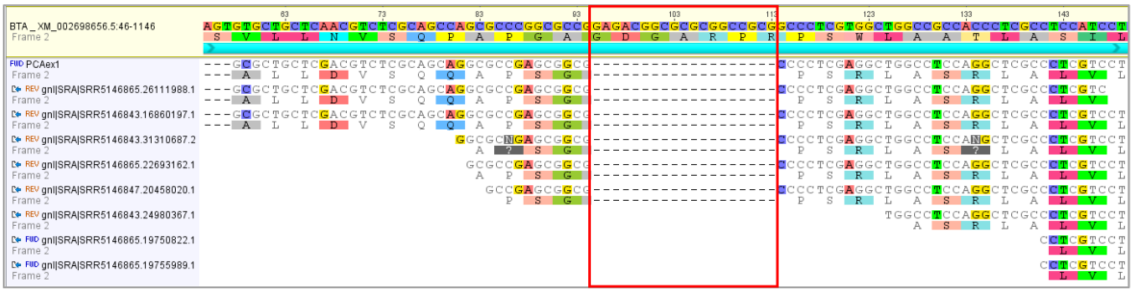

Figure S12: SRA validation for 19 nucleotide deletion in exon 1 of *P. catodon Mtnr1a*.

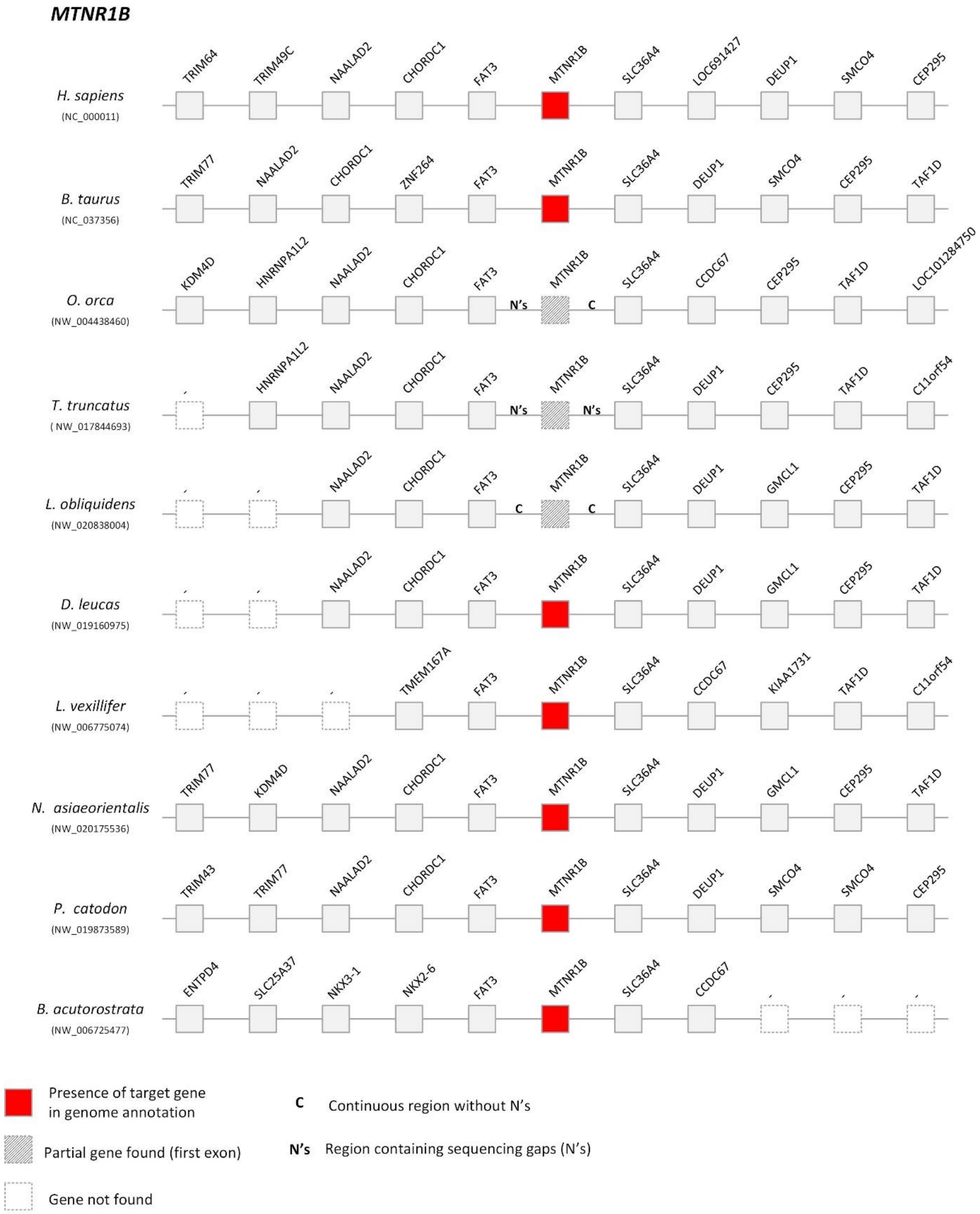

Figure S13: Comparative synteny maps of *Mtnr1b* genomic *locus.*

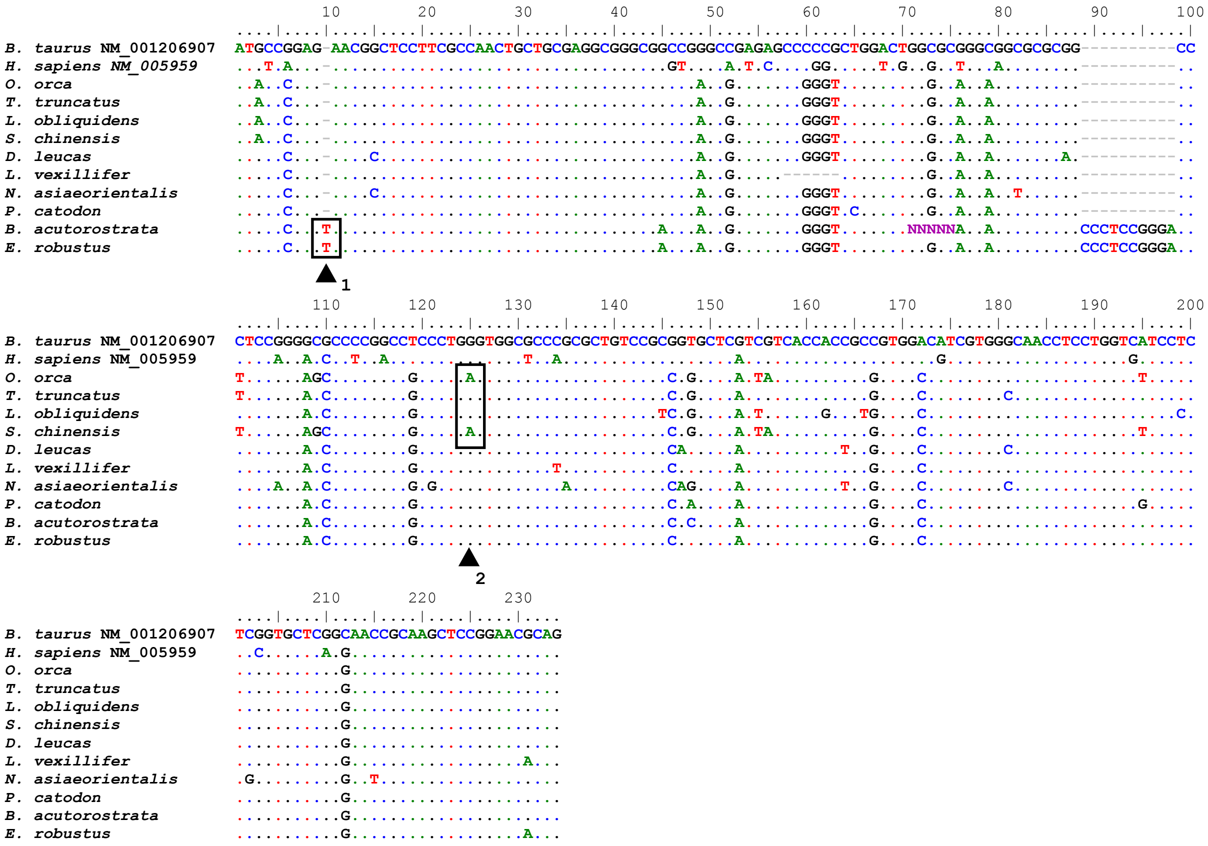

Figure S14: Multiple alignment of the predicted exon 1 of *Mtnr1b* in the listed species. Nucleotide insertion, generating a premature stop codon, in *B. acutorostrata* and *E. robustus* and premature stop codon retrieved in *O. orca* and *S. chinensis*, mutations validated with SRA (when available), are represented in the corresponding position with a black arrow.

Orcinus orca Stop codon exon 1
SRA searched
SRR1164379- University of Durham 2014-02-13 **Sample ID:** SAMN02595096 (AR-Genome)

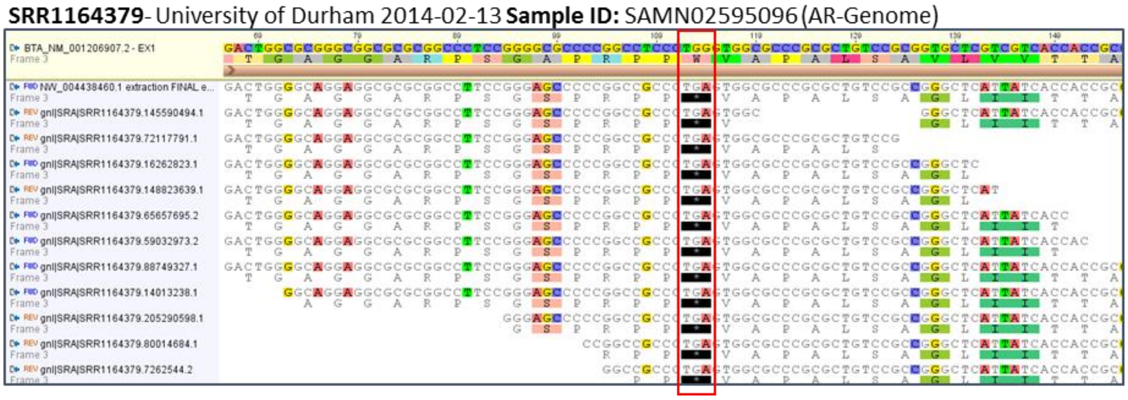

Figure S15: SRA validation for stop mutation (red box) in exon 1 of the *Mntr1b* gene from *O. orca*.

Balaenoptera acutorostrata Exon1 1bp insertion creates premature stop codon
SRA searched
SRR924087- Korea Institute of Ocean Science and Technology 2013-10-31 **Sample ID:** SAMN02192644 **(**MinkeWhale-01)
SRR4011112- Institute of Marine Research 2016-08-13 **Sample ID:** SAMN05447714 (AT)

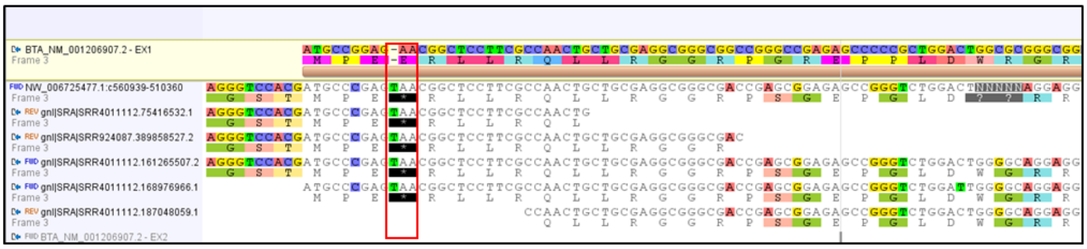

Eschrichtius robustus Exon1 1bp insertion creates premature stop codon
SRA searched
SRR5495100- Purdue University 2017-05-02 **Sample ID:** SAMN06837694 (GFD-02)
SRR5495106- Purdue University 2017-05-02 **Sample ID:** SAMN06837692 (ER-14-168)

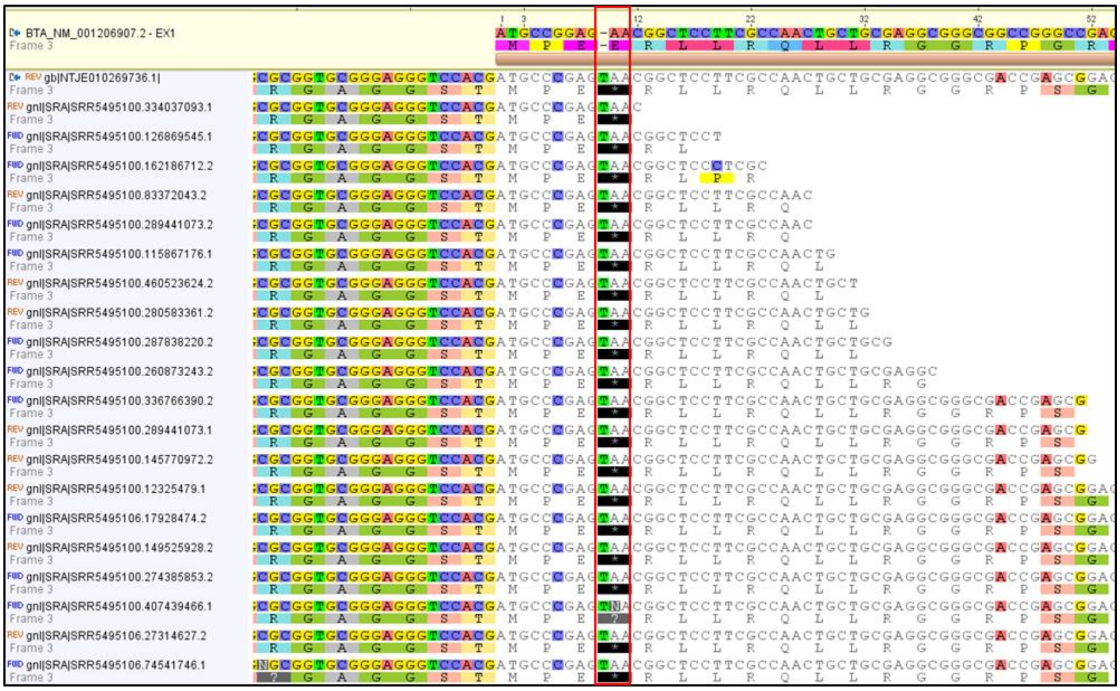

Figure S16: SRA validation for nucleotide insertion generating a premature stop mutation (red box) in exon 1 of the *Mntr1b* gene from *B. acutorostrata* and *E. robustus*.

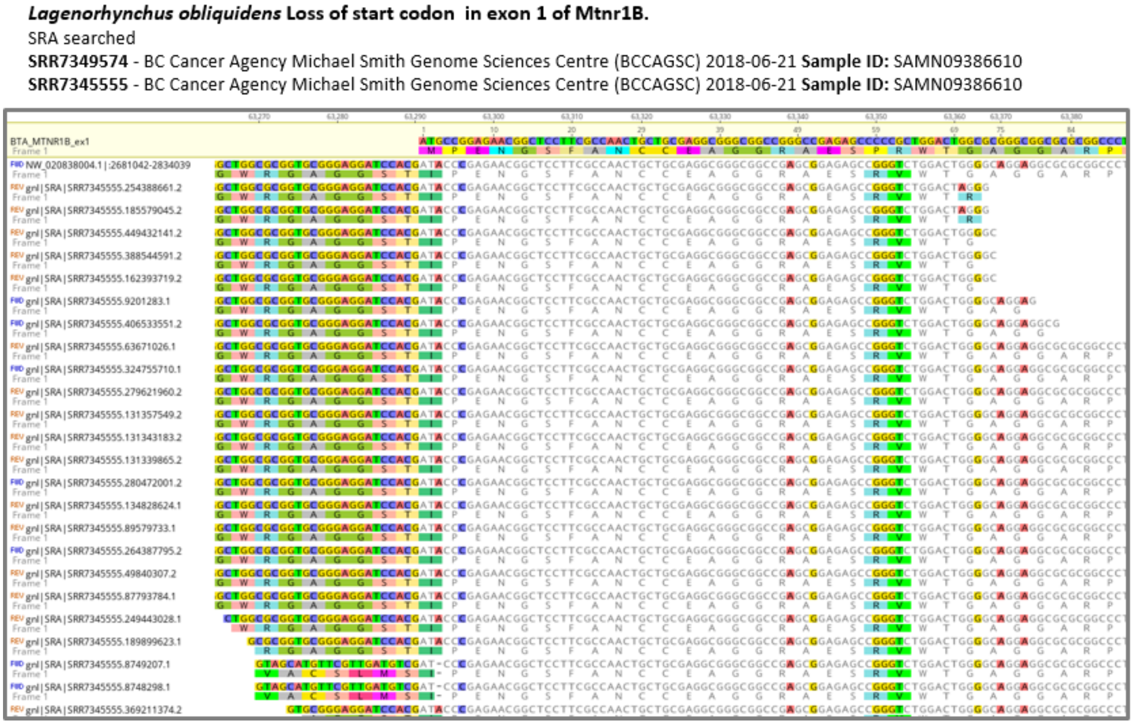

Figure S17: SRA validation for loss of start codon in exon 1 of the *Mntr1b* gene from *L. obliquidens*.

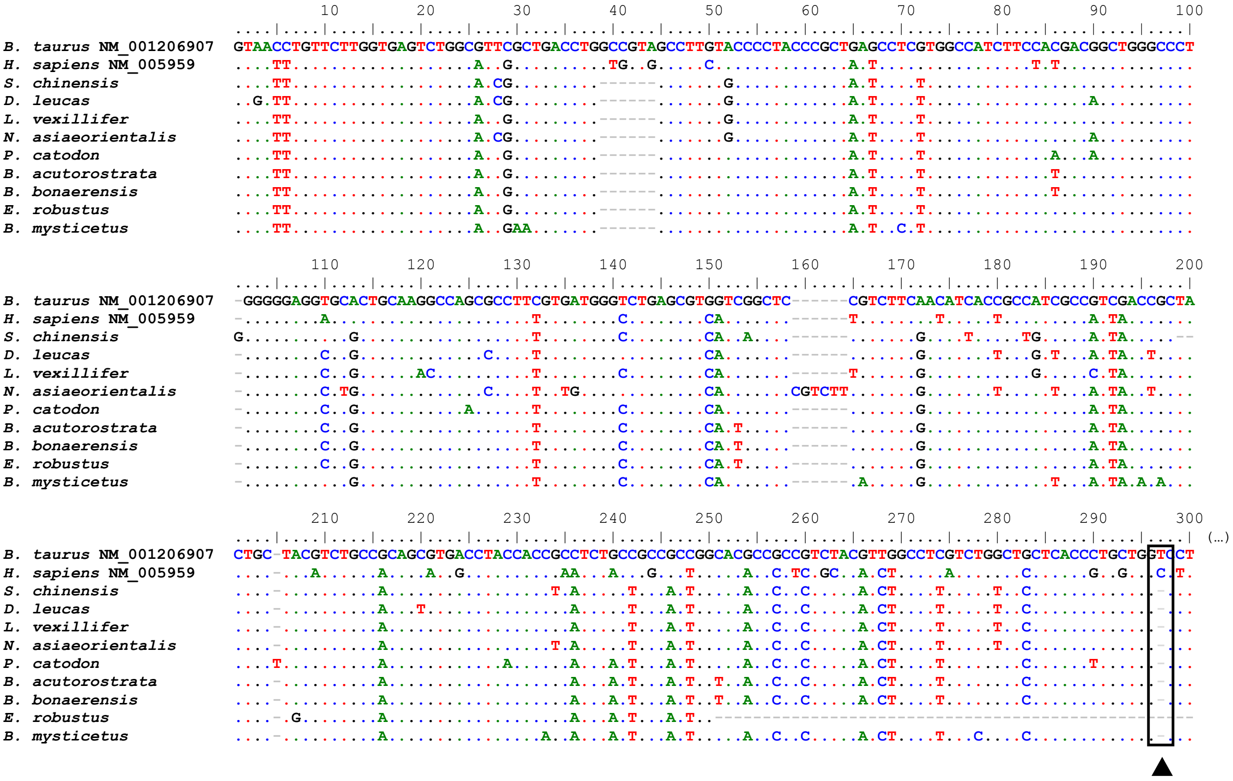

Figure S18: Partial multiple alignment of the predicted exon 2 of *Mtnr1b* in the listed species. Conserved nucleotide deletion, validated with SRA (when available), is represented in the corresponding position with a black arrow.

Delphinapterus leucas conserved 1 nt deletion exon 2

SRA searched

SRR5197962 - BC Cancer Agency Michael Smith Genome Sciences Centre 2017-06-27 **Sample ID:** SAMN06217832 (Qila21)

SRR5659909 - BC Cancer Agency Michael Smith Genome Sciences Centre 2017-06-27 **Sample ID:** SAMN06216270 (Aurora29)

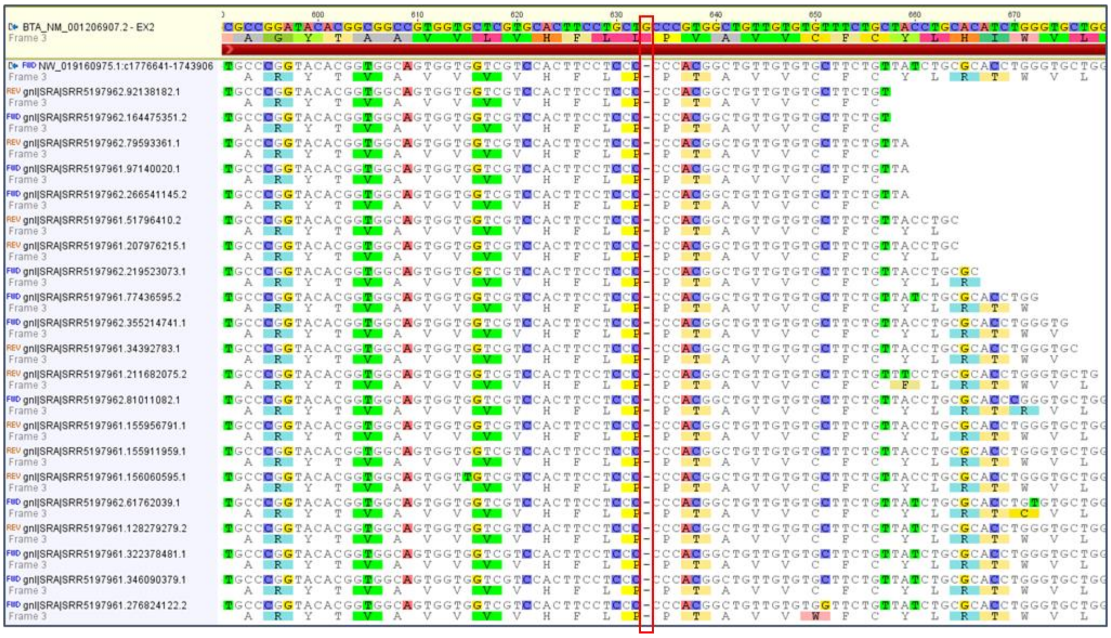

Neophocaena asiaeorientalis conserved 1 nt deletion exon 2

SRA searched

SRR6923630- Northwestern Polytechnical University 2018-04-08 **Sample ID:** SAMN08512128

SRR4292276- Beijing Genome Institute 2017-09-23 **Sample ID:** SAMN05818630

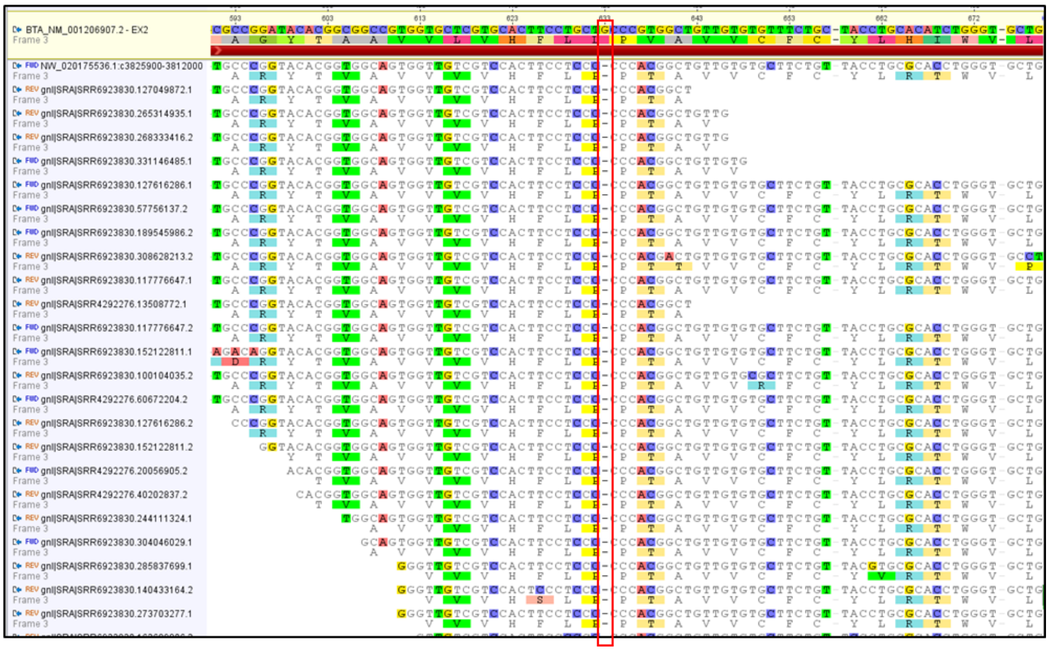

Physeter catodon 1 nt deletion and stop codon exon 2
SRA searched
SRR5146843- The Genome Center at Washington University School of Medicine in St. Louis 2017-01-05 **Sample ID:** SAMN06187413
SRR5146865- The Genome Center at Washington University School of Medicine in St. Louis 2017-01-05 **Sample ID:** SAMN06187411

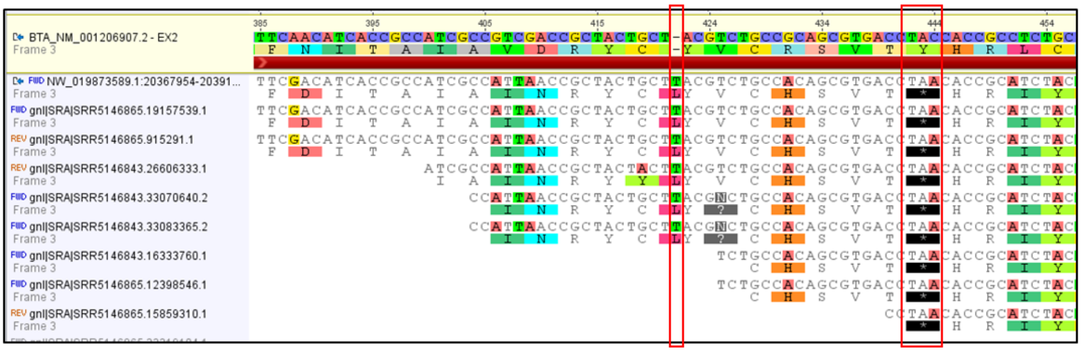

Physeter catodon conserved 1 nt deletion exon 2
SRA searched
SRR5146847- The Genome Center at Washington University School of Medicine in St. Louis 2017-01-05 **Sample ID:** SAMN06187412
SRR5146843- The Genome Center at Washington University School of Medicine in St. Louis 2017-01-05 **Sample ID:** SAMN06187413
SRR5146865- The Genome Center at Washington University School of Medicine in St. Louis 2017-01-05 **Sample ID:** SAMN06187411

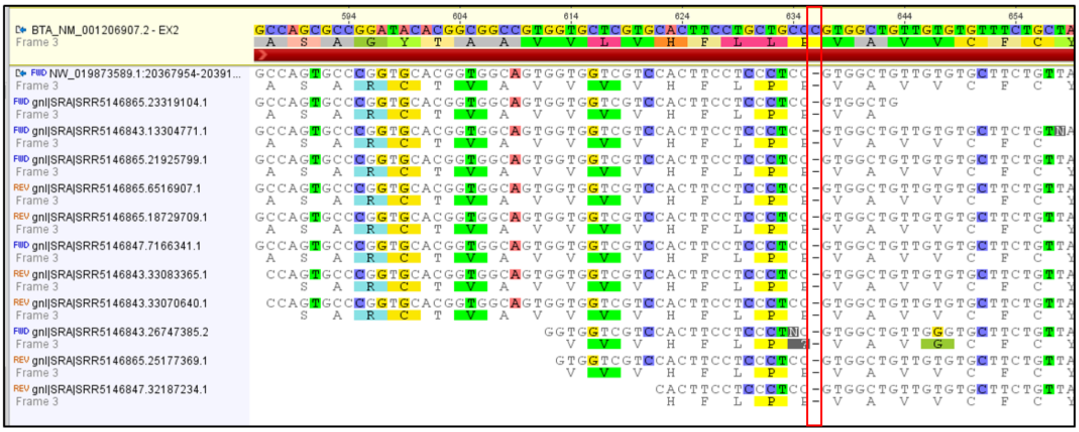

Balaenoptera acutorostrata Conserved 1 nt deletion exon 2
SRA searched
SRR924087- Korea Institute of Ocean Science and Technology 2013-10-31 **Sample ID:** SAMN02192644 **(**MinkeWhale-01)
SRR4011112- Institute of Marine Research 2016-08-13 **Sample ID:** SAMN05447714 (AT)

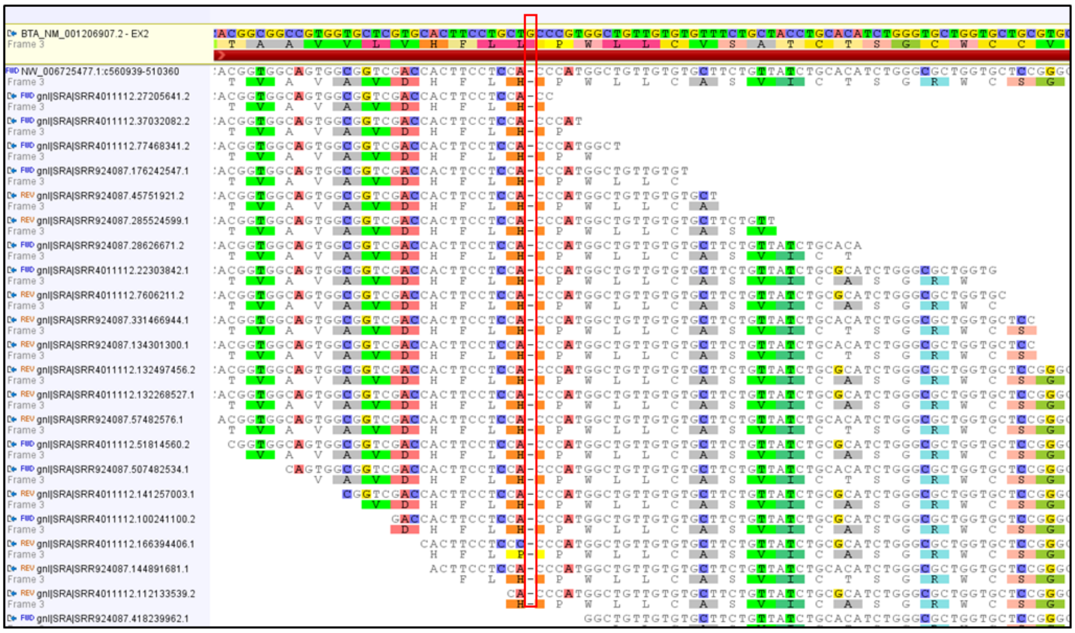

Balaenoptera bonaerensis Conserved 1 nt deletion exon 2

SRA searched

SRR4011114- Institute of Marine Research 2016-08-13 **Sample ID:** SAMN05447715

SRR4011113- Institute of Marine Research 2016-08-13 **Sample ID:** SAMN05447715

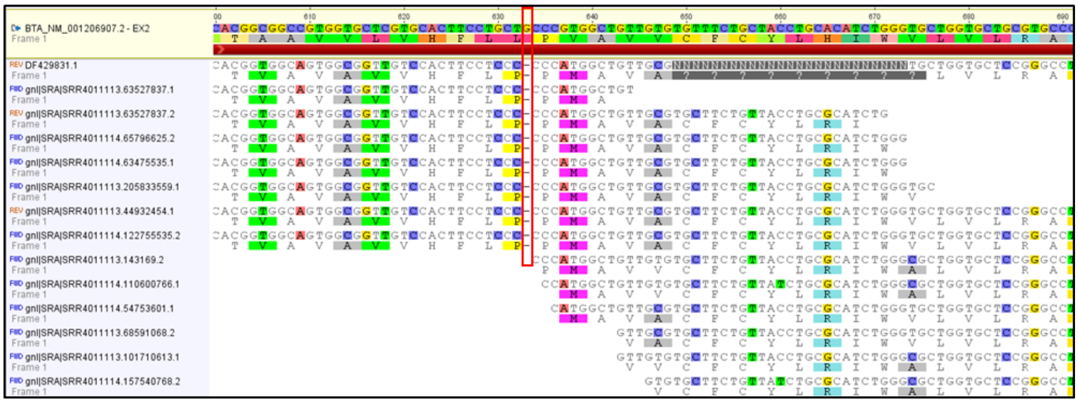

Balaena mysticetus Conserved 1 nt deletion exon 2

SRA searched

SRR1685385- University of Liverpool 2015-01-06 **Sample ID:** SAMN03225705 (Bowhead ID325)

SRR1685386- University of Liverpool 2015-01-06 **Sample ID:** SAMN03225705 (Bowhead ID325)

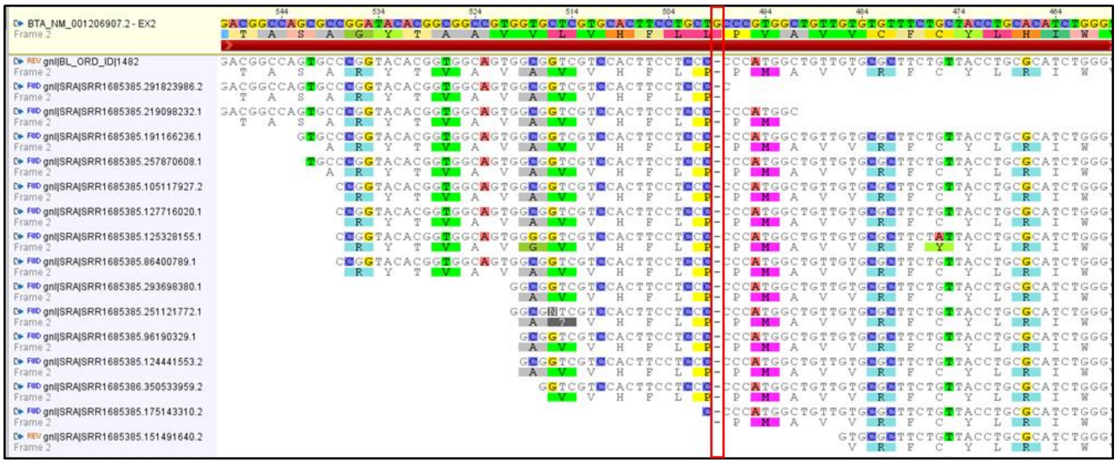

Figure S19: SRA validation for conserved nucleotide deletion in exon 2 of *Mtnr1b* from *D. leucas*, *N. asiaeorientalis*, *P. catodon*, *B. acutorostrata*, *B. bonaerensis* and *B. mysticetus*.

Eschrichtius robustus SRA reads bridging Exon 2 282bp deletion (NNN)
SRA searched
SRR5495100- Purdue University 2017-05-02 **Sample ID:** SAMN06837694 (GFD-02)
SRR5495106- Purdue University 2017-05-02 **Sample ID:** SAMN06837692 (ER-14-168)

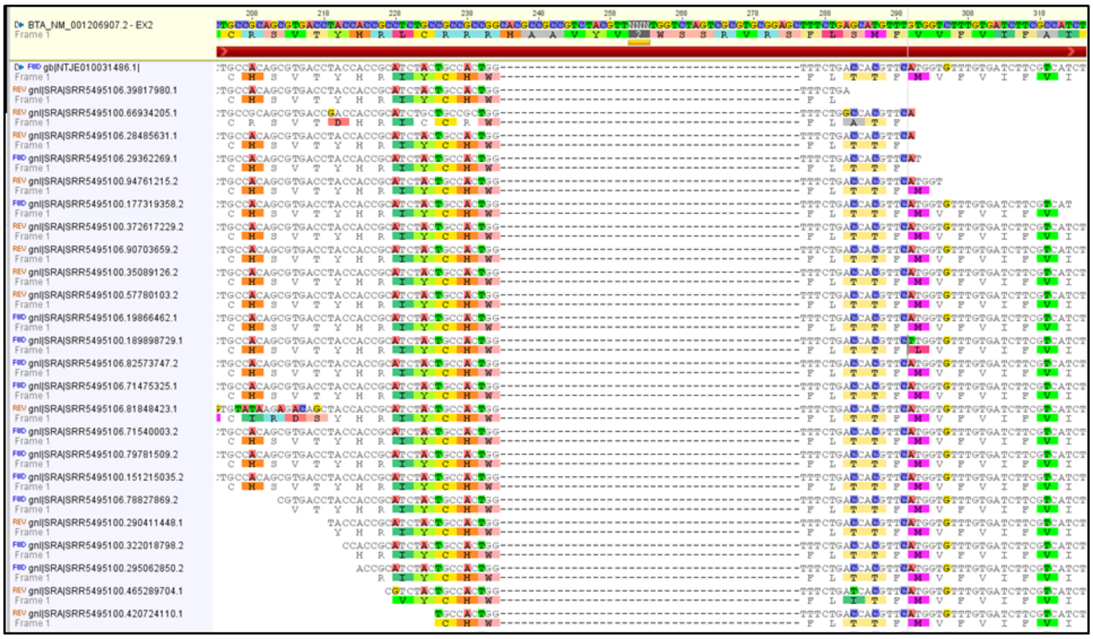

Figure S20: SRA validation for nucleotide deletion in exon 2 of *Mtnr1b* from *E. robustus*.

Hippopotamus amphibius *Asmt* Exon 1 assembly using SRA
SRA searched
WGS
SRR5663648 -Senckenberg Gesellschaft fuer Naturforschung **Released:** 2018-04-05 **Sample:** SAMN07206988
SRR5663647 -Senckenberg Gesellschaft fuer Naturforschung **Released:** 2018-04-05 **Sample:** SAMN07206988
RNA-seq
SRX1164570-SRR2183469 School of Biological & Chemical Sciences, Queen Mary University of London **Released:** 2015-09-30 **Sample:** SAMN04009372

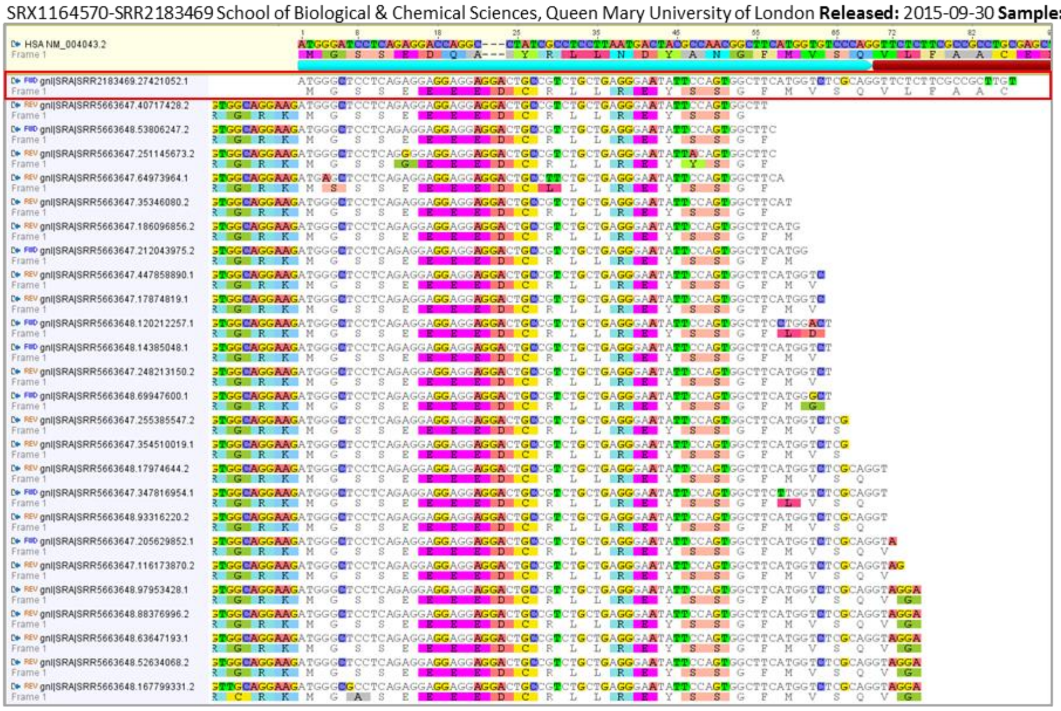

Red box highlights a spliced RNA-Seq read spanning from exon 1 to exon 2

Hippopotamus amphibius *Asmt* Exon 5 assembly using SRA
SRA searched
WGS
SRR5663648 -Senckenberg Gesellschaft fuer Naturforschung **Released:** 2018-04-05 **Sample:** SAMN07206988
SRR5663647 -Senckenberg Gesellschaft fuer Naturforschung **Released:** 2018-04-05 **Sample:** SAMN07206988
SRR5663646- Senckenberg Gesellschaft fuer Naturforschung **Released:** 2018-04-05 **Sample:** SAMN07206988
RNA-seq
SRX1164570-SRR2183469 School of Biological & Chemical Sciences, Queen Mary University of London **Released:** 2015-09-30 **Sample:** SAMN04009372

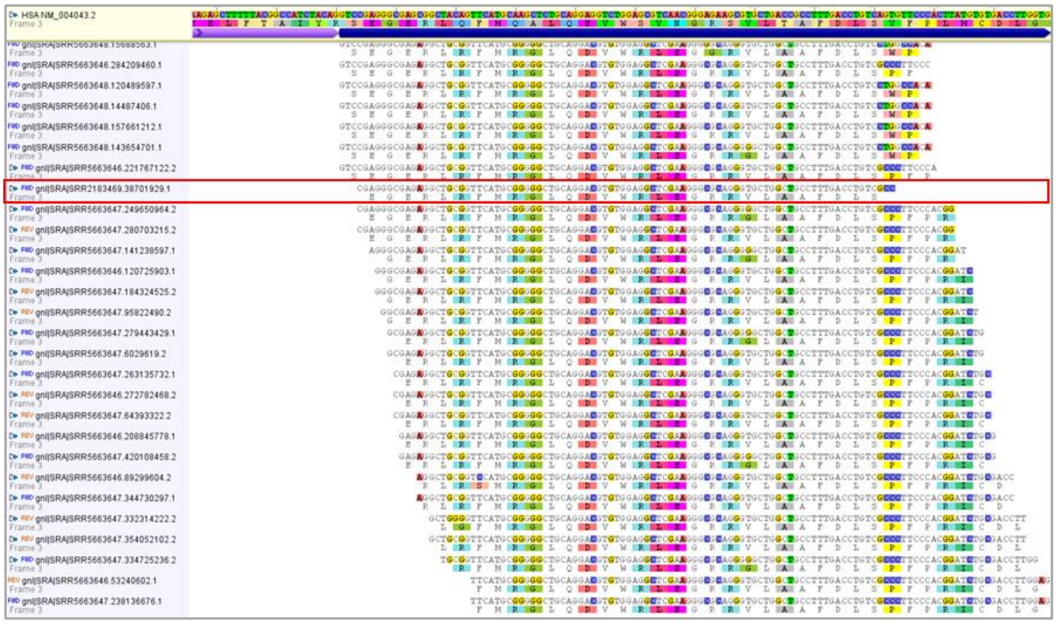

Red box highlights RNA-Seq read mapping to exon 5

Figure S21: *H. amphibius Asmt* exon 1 and 5 assembly.

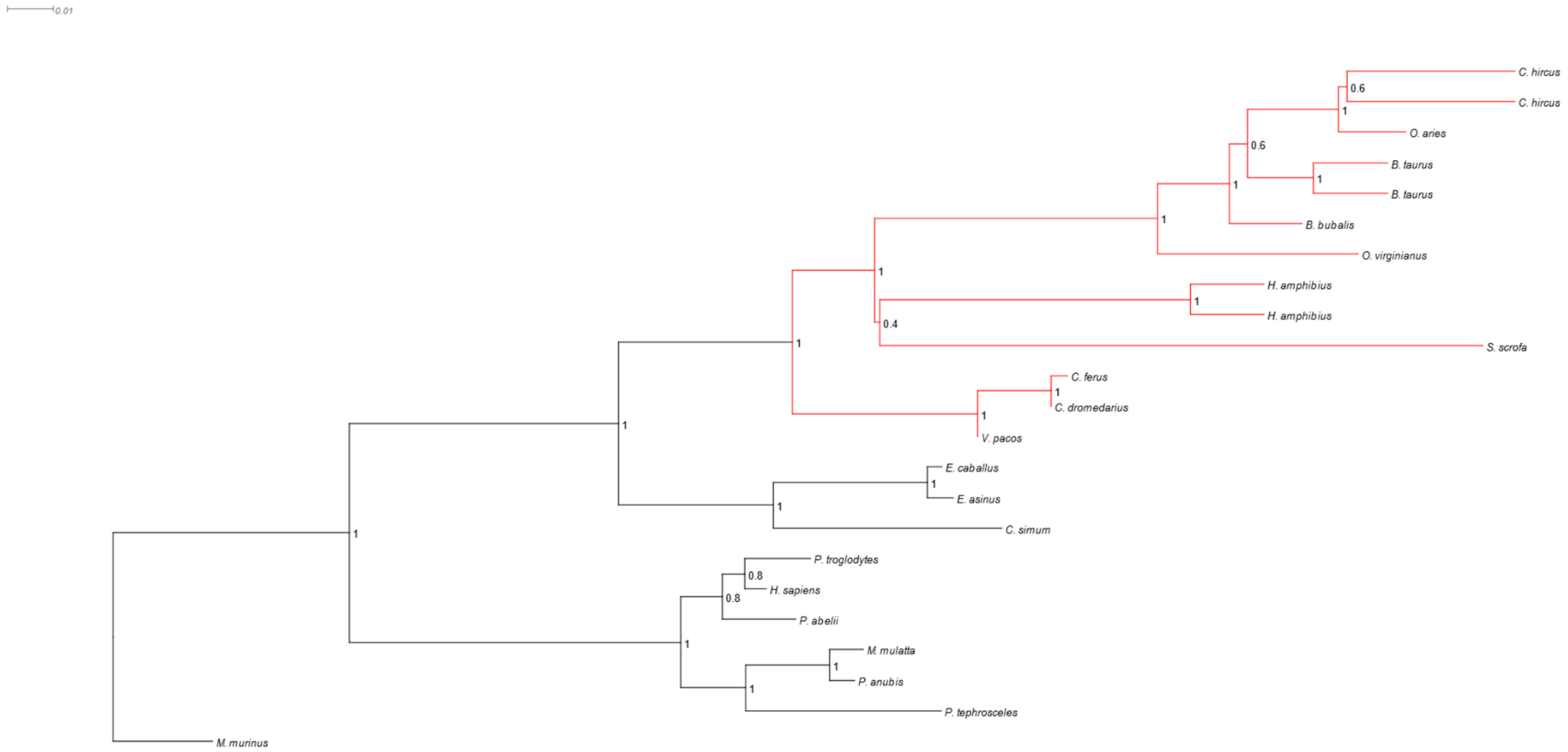

Figure S22: Maximum likelihood phylogenetic analysis of Perissodactyla, Artiodactyla and Primate *Aanat* genes. Node values represent branch support using the aBayes algorithm. In red the clade containing the *H. amphibius*.

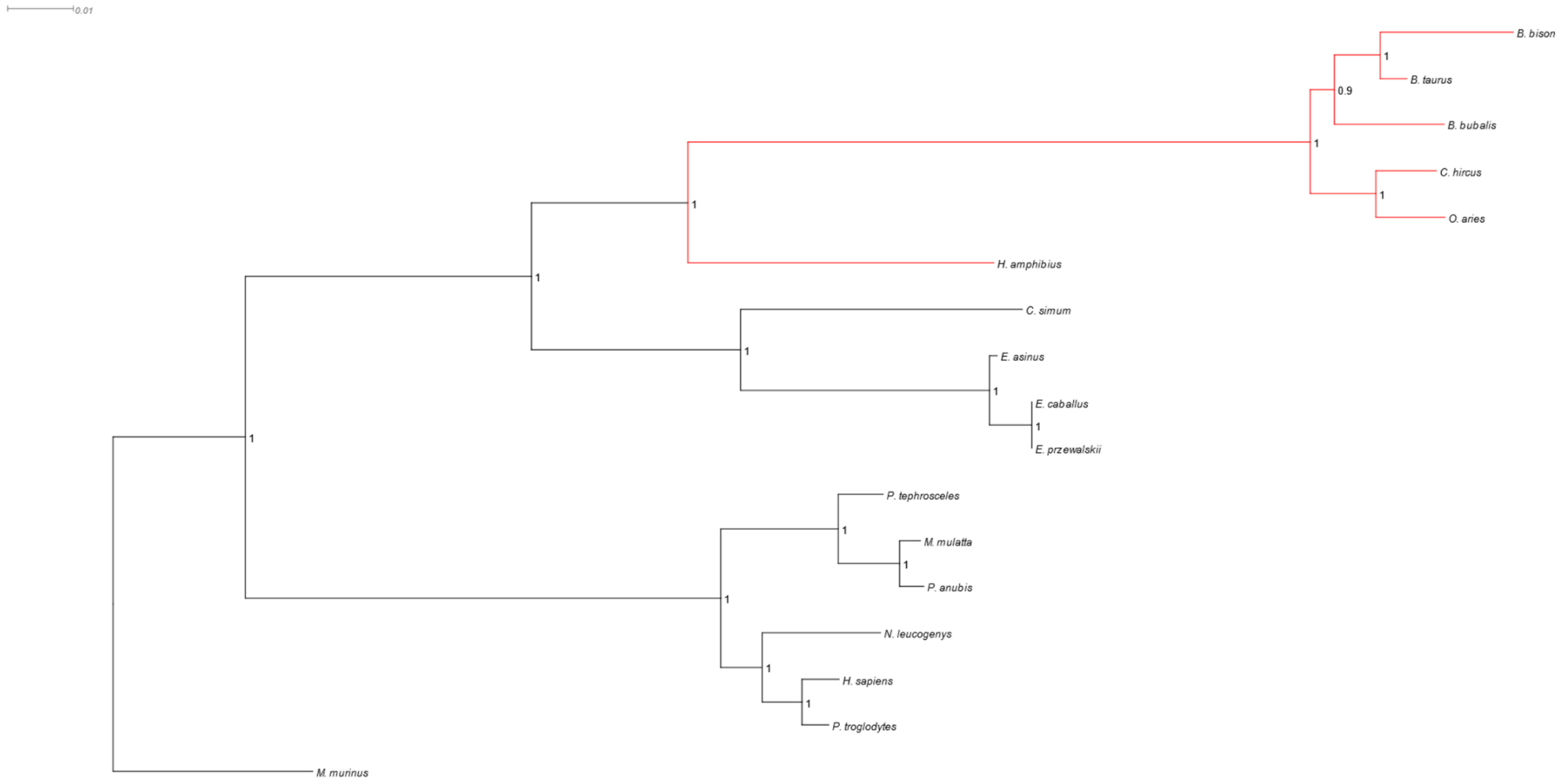

Figure S23: Maximum likelihood phylogenetic analysis of Perissodactyla, Artiodactyla and Primate *Mtnr1a* genes. Node values represent branch support using the aBayes algorithm. In red the clade containing the *H. amphibius*.

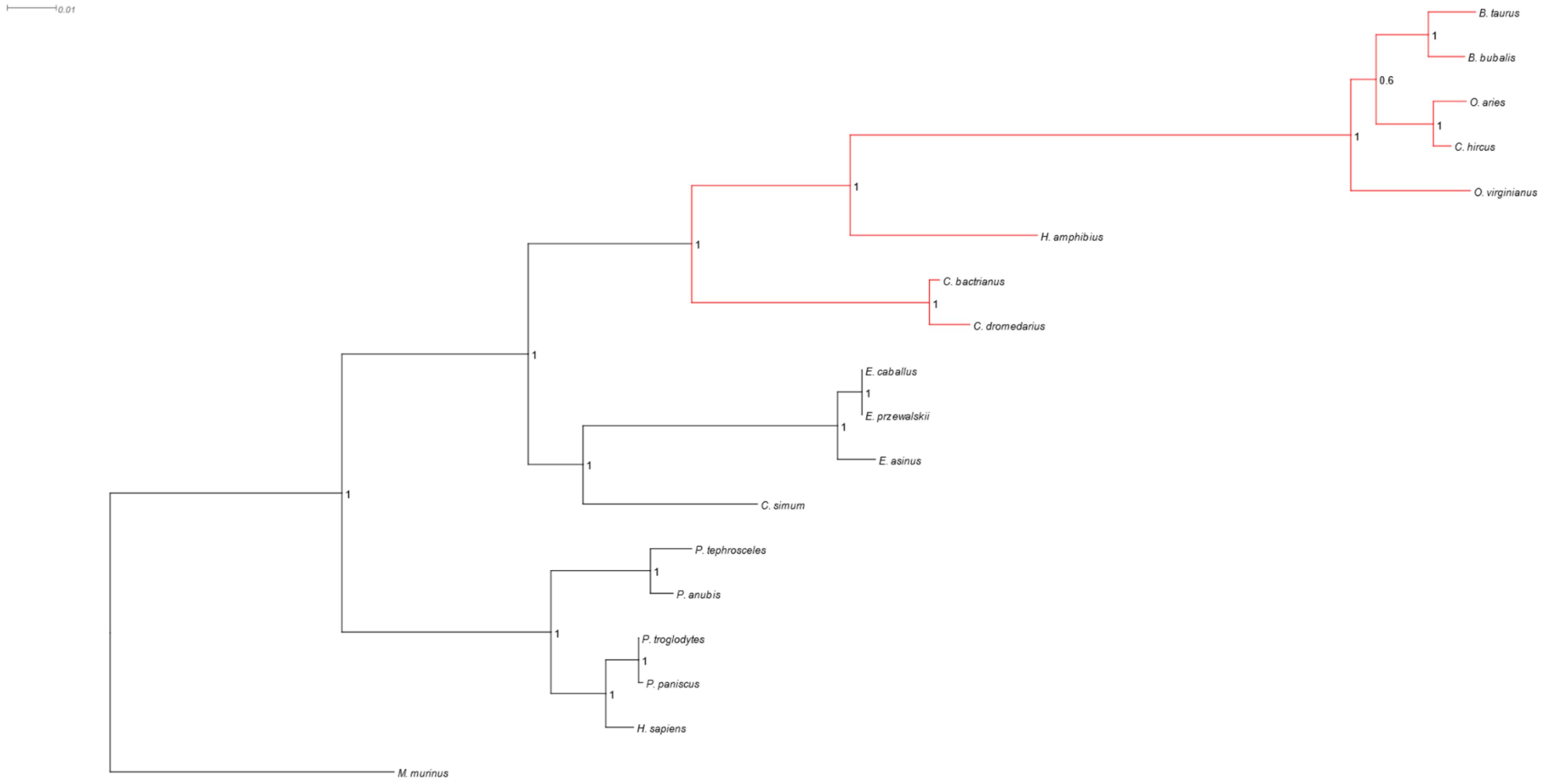

Figure S24: Maximum likelihood phylogenetic analysis of Perissodactyla, Artiodactyla and Primate *Mtnr1b* genes. Node values represent branch support using the aBayes algorithm. In red the clade containing the *H. amphibius*.

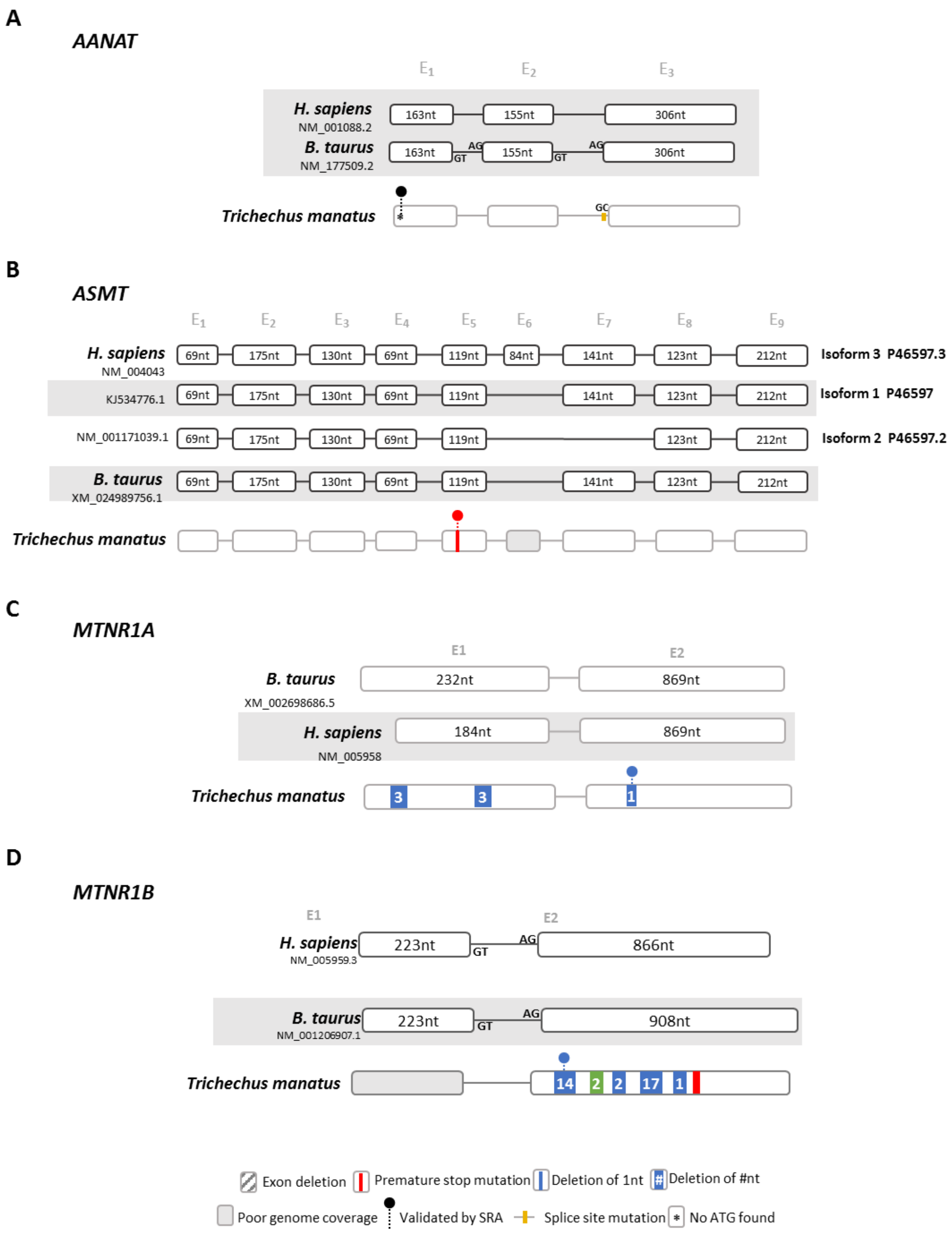

Figure S25: *T. manatus latirostris* gene annotations. Schematic representation of the gene structure of human and B. taurus *Aanat* (A), *Asmt* (B), *Mtnr1a* (C) and *Mtnr1b* (D) genes, each box represents an exon and lines represent intronic region, and sSchematic representation of the corresponding genes identified in *T. manatus latirostris* and location of the identified mutations. Non canonical splice sites are indicated above the corresponding annotation.

Trichechus manatus latirostris
SRA searched:
SRR331137 - Broad Institute (BI) 2011-08-17 **Sample ID:** SAMN00632092
SRR331138 - Broad Institute (BI) 2011-08-17 **Sample ID:** SAMN00632092
SRR331139 - Broad Institute (BI) 2011-08-17 **Sample ID:** SAMN00632092
SRR331142 - Broad Institute (BI) 2011-08-17 **Sample ID:** SAMN00632092

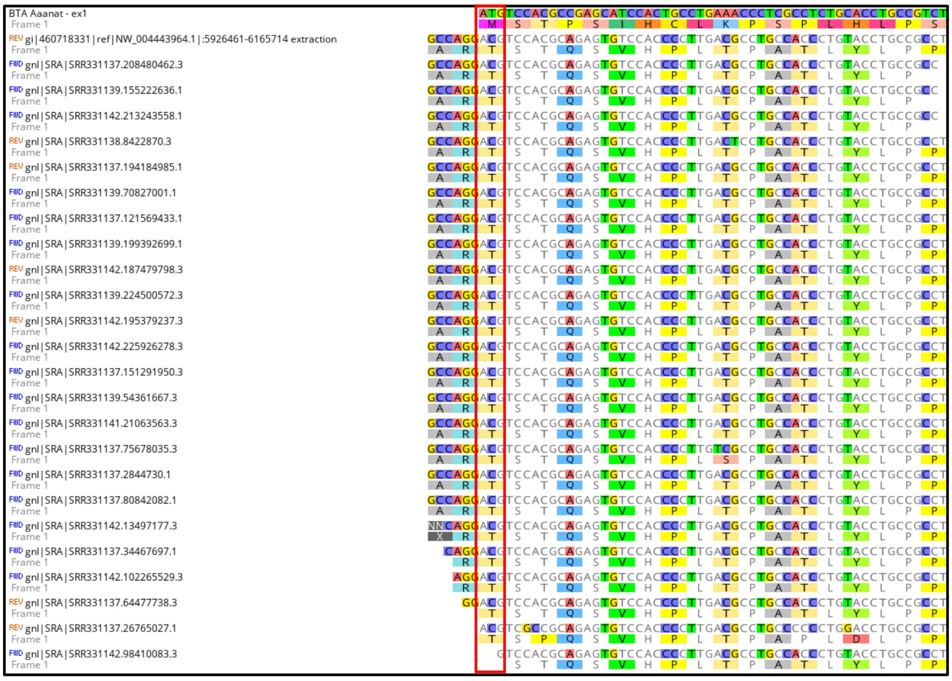

Figure S26: SRA validation for loss of start codon in exon 1 of the *Aanat* gene from *T. manatus latirostris.*

Trichechus manatus latirostris
SRA searched:
SRR331143 - Broad Institute (BI) 2011-08-17 **Sample ID:** SAMN00632092
SRR331145 - Broad Institute (BI) 2011-08-17 **Sample ID:** SAMN00632092
SRR353370 - Broad Institute (BI) 2011-10-16 **Sample ID:** SAMN00632092
SRR353371 - Broad Institute (BI) 2011-10-16 **Sample ID:** SAMN00632092

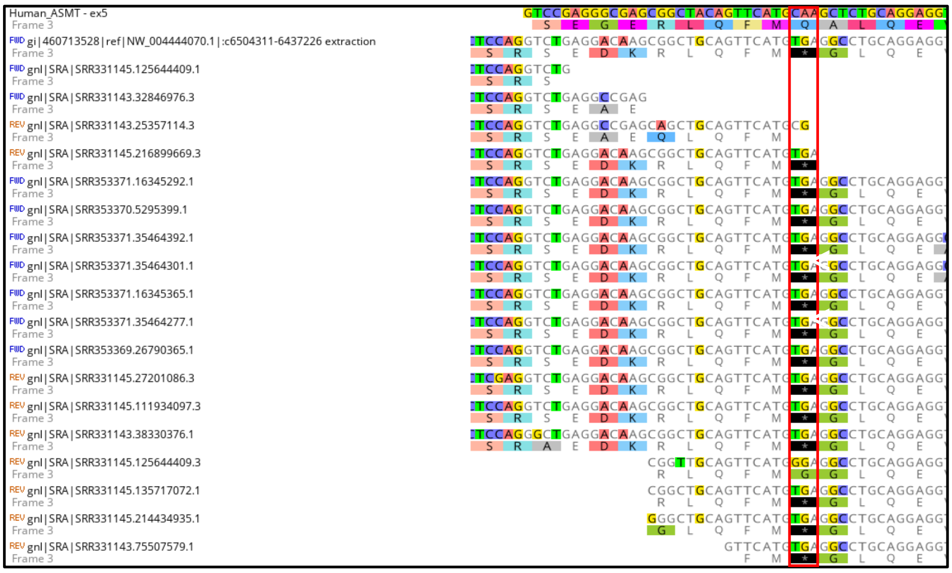

Figure S27: SRA validation for premature stop codon in exon 5 of the *Asmt* gene from *T. manatus latirostris.*

Trichechus manatus latirostris

SRA searched:

SRR307134 - Broad Institute (BI) 2011-07-08 **Sample ID:** SAMN00632092

SRR307135 - Broad Institute (BI) 2011-07-08 **Sample ID:** SAMN00632092

SRR307136 - Broad Institute (BI) 2011-07-08 **Sample ID:** SAMN00632092

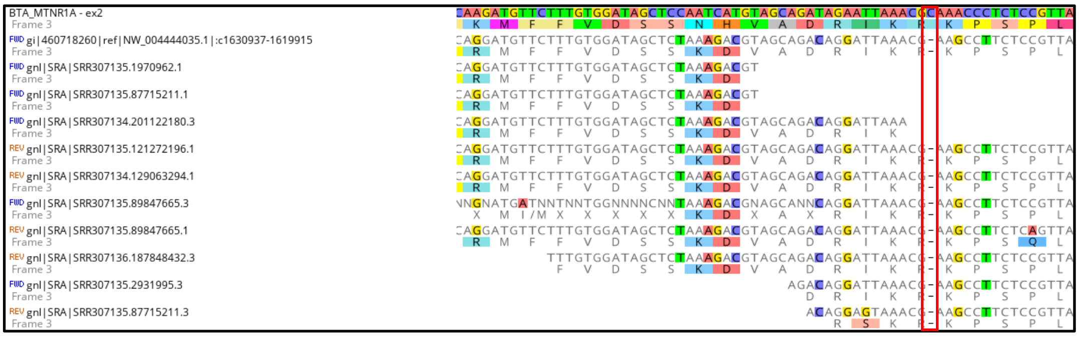

Figure S28: SRA validation for single nucleotide insertion in exon 2 of the *Mtnr1a* gene from *T. manatus latirostris.*

Trichechus manatus latirostris

SRA searched:

SRR307134 - Broad Institute (BI) 2011-07-08 **Sample ID:** SAMN00632092

SRR307135 - Broad Institute (BI) 2011-07-08 **Sample ID:** SAMN00632092

SRR307136 - Broad Institute (BI) 2011-07-08 **Sample ID:** SAMN00632092

SRR307137 - Broad Institute (BI) 2011-07-08 **Sample ID:** SAMN00632092

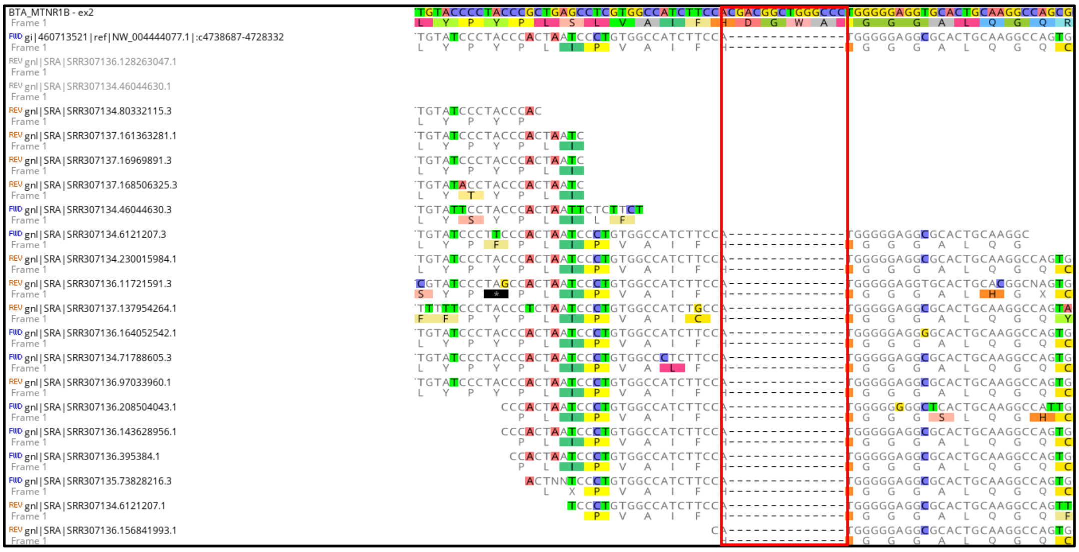

Figure S29: SRA validation for frameshift mutation in exon 2 of the *Mtnr1b* gene from *T.manatus latirostris.*

Table S1: Blast-n output of *Bos taurus* and *Homo sapiens* sequences against the *H. amphibius* genome. These values were used to find the loci of *Hippopotamus amphibius* that contains the genes *Aanat*, *Mtnr1a* and *Mtnr1b*.

Species	Accession numbers	Gene symbol	Scaffolds of *H. amphibius* genome	ID%	Exon number	Length	Gaps	qstart	qend	sstart	send	E-value	Bitscore
Bos taurus	NM_177509.2	AANAT	NKPW01005924.1	87.117	1	163	0	1	163	56196	56034	1.71e-44	185
Bos taurus	NM_177509.2	AANAT	NKPW01005924.1	91.720	2	157	0	163	319	55714	55558	1.68e-54	219
Bos taurus	NM_177509.2	AANAT	NKPW01005924.1	86.452	3	310	2	316	624	55168	54860	1.24e-90	339
Bos taurus	XM_024981048.1	AANAT	NKPW01005924.1	87.730	1	163	0	1	163	44541	44379	3.66e-46	191
Bos taurus	XM_024981048.1	AANAT	NKPW01005924.1	90.604	2	149	0	163	311	44059	43911	2.19e-48	198
Bos taurus	XM_024981048.1	AANAT	NKPW01005924.1	83.495	3	309	0	316	624	43518	43210	1.26e-75	289
Bos taurus	NM_001206907.2	MTNR1B	NKPW01013551.1	94.667	1	225	0	1	225	40814	40590	1.06e-93	350
Bos taurus	NM_001206907.2	MTNR1B	NKPW01013551.1	88.575	2	814	0	221	1034	26808	25995	0.0	989
Bos taurus	NM_005958.4	MTNR1A	NKPW01072562.1	84.737	1	190	6	5	185	789	978	3.79e-43	182
Bos taurus	NM_005958.4	MTNR1A	NKPW01009466.1	85.682	2	873	3	183	1053	13832	14702	0.0	917
Homo sapiens	NM_004043.2	ASMT	NKPW01039339.1	81.250	2	176	2	68	241	2180	2005	6,87E-29	141
Homo sapiens	NM_004043.2	ASMT	NKPW01075055.1	78.333	5	120	0	441	560	1215	1334	5,46E-10	78.7
Homo sapiens	NM_004043.2	ASMT	NKPW01040565.1	77.778	7	144	1	653	795	6591	6734	9,08E-13	87.9
Homo sapiens	NM_004043.2	ASMT	NKPW01040565.1	86.667	8	135	3	786	918	8397	8530	1,48E-30	147
Homo sapiens	NM_004043.2	ASMT	NKPW01040565.1	74.528	9	212	2	908	1118	2399	2609	7,02E-14	91.6

Table S2: Accession numbers of the *Aanat* orthologues sequences used in phylogenetic analyses.

Species	Tree CODE	Gene ID	Accession Number Nucleotide	Gene Symbol	Lineage
Pan troglodytes	*P. troglodytes*	503504	NM_001012440.1	Aanat	Primate-Hominoidae
Homo sapiens	*H. sapiens*	15	NM_001166579.1	Aanat	Primate-Hominoidae
Pongo abelii	*P. abelii*	100433977	XM_002827870.4	Aanat	Primate-Hominoidae
Macaca mulatta	*M. mulatta*	706924	NM_001047127.1	Aanat	Primate-Cercopithecoidea
Papio anubis	*P. anubis*	101021111	XM_021928021.1	Aanat	Primate-Cercopithecoidea
Piliocolobus tephrosceles	*P. tephrosceles*	111542384	XM_023211738.1	Aanat	Primate-Cercopithecoidea
Microcebus murinus	*M. murinus*	105878976	XM_012778898.2	Aanat	Primate-Strepsirrhini
Bubalus bubalis	*B. bubalis*	102412521	XM_006045309.1	LOC102412521	Artiodactyla
Bos taurus	*B. taurus*	281583	NM_177509.2	Aanat	Artiodactyla
Bos taurus	*B. taurus*	112442710	XM_024981048.1	Aanat	Artiodactyla
Capra hircus	*C. hircus*	102190991	XM_018064062.1	Aanat	Artiodactyla
Capra hircus	*C. hircus*	100860947	NM_001285561.1	Aanat	Artiodactyla
Ovis aries	*O. aries*	443531	NM_001009461.1	Aanat	Artiodactyla
Odocoileus virginianus texanus	*O. virginianus*	110152065	XM_020915599.1	LOC110152065	Artiodactyla
Hippopotamus amphibius	*H. amphibius*	-	-	-	Artiodactyla
Hippopotamus amphibius	*H. amphibius*	-	-	-	Artiodactyla
Sus scrofa	*S. scrofa*	100518330	XM_005656910.3	LOC100518330	Artiodactyla
Vicugna pacos	*V. pacos*	102540360	XM_006199417.1	Aanat	Artiodactyla
Camelus dromedarius	*C. dromedarius*	105092185	XM_010983838.1	Aanat	Artiodactyla
Camelus ferus	*C. ferus*	102522698	XM_006176501.1	Aanat	Artiodactyla
Equus asinus	*E. asinus*	106830333	XM_014839904.1	Aanat	Perissodactyla-Equidea
Equus caballus	*E. caballus*	100058770	XM_023651967.1	Aanat	Perissodactyla-Equidea
Ceratotherium simum simum	C. simum	101404984	XM_014789296.1	LOC101404984	Perissodactyla-Rhinoceratidae

Table S3: Accession numbers of the *Mtnr1a* orthologues sequences used in phylogenetic analyses.

**Species**	**Tree CODE**	**Gene ID**	**Accession Number Nucleotide**	**Gene Symbol**	**Lineage**
Homo sapiens	*H. sapiens*	4543	NM_005958.4	MTNR1A	Primate-Hominoidae
Nomascus leucogenys	*N. leucogenys*	100594737	XM_003271501.1	MTNR1A	Primate-Hominoidae
Pan troglodytes	*P. troglodytes*	471417	XM_016952653.1	MTNR1A	Primate-Hominoidae
Macaca mulatta	*M. mulatta*	702686	XM_001090972.3	MTNR1A	Primate-Cercopithecoidea
Papio anubis	*P. anubis*	101005602	XM_003899434.4	MTNR1A	Primate-Cercopithecoidea
Piliocolobus tephrosceles	*P. tephrosceles*	111548519	XM_023221040.2	MTNR1A	Primate-Cercopithecoidea
Microcebus murinus	*M. murinus*	105882472	XM_012784869.1	MTNR1A	Primate-Strepsirrhini
Bison bison bison	*B. bison*	104985582	XM_010835821.1	MTNR1A	Artiodactyla
Bubalus bubalis	*B. bubalis*	102415439	XM_006076173.1	MTNR1A	Artiodactyla
Bos taurus	*B. taurus*	539948	XM_002698656.4	MTNR1A	Artiodactyla
Capra hircus	*C. hircus*	102189094	XM_018041838.1	MTNR1A	Artiodactyla
Ovis aries	*O. aries*	443022	NM_001009725.1	MTNR1A	Artiodactyla
Hippopotamus amphibius	*H. amphibius*	-	-	MTNR1A	Artiodactyla
Equus caballus	*E. caballus*	100056423	XM_001490171.3	MTNR1A	Perissodactyla-Equidea
Equus przewalskii	*E. przewalskii*	103549958	XM_008518624.1	MTNR1A	Perissodactyla-Equidea
Equus asinus	*E. asinus*	106840379	XM_014855805.1	MTNR1A	Perissodactyla-Equidea
Ceratotherium simum simum	C. simum	101391490	XM_004428772.1	LOC101391490	Perissodactyla-Rhinoceratidae

Table S4: Accession numbers of the *Mtnr1b* orthologues sequences used in phylogenetic analyses.

Species	Tree CODE	Gene ID	Accession Number Nucleotide	Gene Symbol	Lineage
Pan troglodytes	*P. troglodytes*	466747	XM_016921786.2	Mtnr1b	Primate-Hominoidae
Homo sapiens	*H. sapiens*	4544	NM_005959.3	Mtnr1b	Primate-Hominoidae
Pan paniscus	*P. paniscus*	100973621	XM_003813777.1	Mtnr1b	Primate-Hominoidae
Papio anubis	*P. anubis*	100999162	XM_003910543.3	Mtnr1b	Primate-Cercopithecoidea
Piliocolobus tephrosceles	*P. tephrosceles*	111540700	XM_023209139.2	Mtnr1b	Primate-Cercopithecoidea
Microcebus murinus	*M. murinus*	105857333	XM_012739636.1	Mtnr1b	Primate-Strepsirrhini
Ovis aries	*O. aries*	100174786	NM_001130938.1	Mtnr1b	Artiodactyla
Capra hircus	*C. hircus*	102176302	XM_018042917.1	Mtnr1b	Artiodactyla
Bubalus bubalis	*B. bubalis*	102409257	XM_006053939.1	Mtnr1b	Artiodactyla
Bos taurus	*B. taurus*	528665	NM_001206907.2	Mtnr1b	Artiodactyla
Hippopotamus amphibius	*H. amphibius*	-	-	Mtnr1b	Artiodactyla
Camelus bactrianus	*C. bactrianus*	105083508	XM_010973367.1	Mtnr1b	Artiodactyla
Camelus dromedarius	*C. dromedarius*	105094616	XM_010986681.1	Mtnr1b	Artiodactyla
Odocoileus virginianus texanus	*O. virginianus*	110127734	XM_020878096.1	Mtnr1b	Artiodactyla
Equus caballus	*E. caballus*	100059172	XM_001917051.2	Mtnr1b	Perissodactyla-Equidea
Equus przewalskii	*E. przewalskii*	103548037	XM_008515596.1	Mtnr1b	Perissodactyla-Equidea
Equus asinus	*E. asinus*	106827482	XM_014835349.1	Mtnr1b	Perissodactyla-Equidea
Ceratotherium simum simum	C. simum	101399557	XM_004427448.1	LOC101399557	Perissodactyla-Rhinoceratidae

Gene annotations: In silico gene annotations of *Aanat*, *Mtnr1a* and *Mtnr1b* in *H. amphibius*

Aanat genes of Hippopotamus amphibius annotated by Augustus Software.

----- prediction on sequence number 1 (length = 65446, name = NKPW01005924.1) -----

Gene 1

NKPW01005924.1 AUGUSTUS gene 43218 44549 0.28 - . g2

NKPW01005924.1 AUGUSTUS transcript 43218 44549 0.28 - . g2.t1

NKPW01005924.1 AUGUSTUS stop_codon 43218 43220 . - 0 transcript_id "g2.t1"; gene_id "g2";

NKPW01005924.1 AUGUSTUS terminal 43218 43503 0.38 - 1 transcript_id "g2.t1"; gene_id "g2";

NKPW01005924.1 AUGUSTUS internal 43916 44066 0.37 - 2 transcript_id "g2.t1"; gene_id "g2";

NKPW01005924.1 AUGUSTUS initial 44387 44549 0.8 - 0 transcript_id "g2.t1"; gene_id "g2";

NKPW01005924.1 AUGUSTUS CDS 43218 43503 0.38 - 1 transcript_id "g2.t1"; gene_id "g2";

NKPW01005924.1 AUGUSTUS CDS 43916 44066 0.37 - 2 transcript_id "g2.t1"; gene_id "g2";

NKPW01005924.1 AUGUSTUS CDS 44387 44549 0.8 - 0 transcript_id "g2.t1"; gene_id "g2";

NKPW01005924.1 AUGUSTUS start_codon 44547 44549 . - 0 transcript_id "g2.t1"; gene_id "g2";

coding sequence =
[atgtccacacagaccatccaatacatgaagcctgtggctctgcacctgccacctgggatcccagagtcgccaagccgccagcggcgccataca
ctccccgccaatgagttccgctgcctcaccccagaggacgctgctgacgtgtttgagattgagcgagaggccttcatctctgtctcgggcgcctgc
cccctgcaactggaccaggtccagcacttcctgaccgtgtgtcccgagctgtccctgggctgcttcgtggagggccgcctcgtggccttcatcatt
ggctccctgtgggacaaggagagactcaggcccgggggccacacagcccacctgcacttgctggccgtgcgccgccccttccggcgtctggg
caacggttccaccctgctctggcgctctctgcaccacctggatggcaagccggccgtgcgccggacctcgctcatgtgcgaggacccattggtgc
ccttctaccagaggttcggcttccaccctgtgggcccgtgtgccgtgaccgtgggctccctcaccttcacggagatggagcgctccccgtggggc
catgcctccctgcgcaggaacagtgacagctga]

protein sequence =
[MSTQTIQYMKPVALHLPPGIPESPSRQRRHTLPANEFRCLTPEDAADVFEIEREAFISVSGACP
LQLDQVQHFLTVCPELSLGCFVEGRLVAFIIGSLWDKERLRPGGHTAHLHLLAVRRPFRRLG
NGSTLLWRSLHHLDGKPAVRRTSLMCEDPLVPFYQRFGFHPVGPCAVTVGSLTFTEMERSP
WGHASLRRNSDS]

Gene 2

NKPW01005924.1 AUGUSTUS gene 54868 56204 0.87 - . g3

NKPW01005924.1 AUGUSTUS transcript 54868 56204 0.87 - . g3.t1

NKPW01005924.1 AUGUSTUS stop_codon 54868 54870 . - 0
transcript_id "g3.t1"; gene_id "g3";

NKPW01005924.1 AUGUSTUS terminal 54868 55173 0.93 - 0
transcript_id "g3.t1"; gene_id "g3";

NKPW01005924.1 AUGUSTUS internal 55567 55721 0.97 - 2
transcript_id "g3.t1"; gene_id "g3";

NKPW01005924.1 AUGUSTUS initial 56042 56204 0.95 - 0 transcript_id "g3.t1"; gene_id "g3";

NKPW01005924.1 AUGUSTUS CDS 54868 55173 0.93 - 0 transcript_id "g3.t1"; gene_id "g3";

NKPW01005924.1 AUGUSTUS CDS 55567 55721 0.97 - 2 transcript_id "g3.t1"; gene_id "g3";

NKPW01005924.1 AUGUSTUS CDS 56042 56204 0.95 - 0 transcript_id "g3.t1"; gene_id "g3";

NKPW01005924.1 AUGUSTUS start_codon 56202 56204 . - 0
transcript_id "g3.t1"; gene_id "g3";

coding sequence =
[atgtccacacagaccatccaatacatgaagcctgtggctctgcacctgccacctgggatcccagagtcgccaagccgccagcggcgccataca
ctccccgccaatgagttccgctgcctcaccccagaggacgctgctgacgtgtttgagattgagcgagaggccttcatctctgtctcgggcgtctgcc
ccctgcaactggaccaggtccagcacttcctgaccgtgtgtcccgagctgtccctgggctgcttcgtggagggccgcctcgtggccttcatcattg
gctccctgtgggacaaggagagactcactcaggagtcactgacactacacaggcccgggggccacacagcccacctgcacttgctggccgtgc
accgcaccttccggcagctggggaagggctccaccctgctctggcgttacctgcaccatctggatggccagccggccgtgcgccgggccgtgc
tcatgtgcgaggacccattggtgcccttctaccagaggttcggcttccaccctgttggcctgtgtgccgcgaccgtgggctccctcaccttcacgga
gatgcaatgctccctgaggggccacgcctccctgcgcagaaacagtgacagctga]

protein sequence =
[MSTQTIQYMKPVALHLPPGIPESPSRQRRHTLPANEFRCLTPEDAADVFEIEREAFISVSGVCP
LQLDQVQHFLTVCPELSLGCFVEGRLVAFIIGSLWDKERLTQESLTLHRPGGHTAHLHLLAV
HRTFRQLGKGSTLLWRYLHHLDGQPAVRRAVLMCEDPLVPFYQRFGFHPVGLCAATVGSL
TFTEMQCSLRGHASLRRNSDS]

Mtnr1a gene of Hippopotamus amphibius annotated by Augustus Software.

----- prediction on sequence number 1 (length = 65443, name = NKPW01072562.1 and NKPW01009466.1) -----

#

Constraints/Hints:

(none)

Predicted genes for sequence number 1 on both strands

start gene g1

NKPW01072562.1_NKPW01009466.1 AUGUSTUS gene 770 16959 0.32 + . g1

NKPW01072562.1_NKPW01009466.1 AUGUSTUS transcript 770 16959 0.32 + . g1.t1

NKPW01072562.1_NKPW01009466.1 AUGUSTUS start_codon 770 772 . + 0 transcript_id "g1.t1"; gene_id "g1";

NKPW01072562.1_NKPW01009466.1 AUGUSTUS initial 770 998 1 + 0 transcript_id "g1.t1"; gene_id "g1";

NKPW01072562.1_NKPW01009466.1 AUGUSTUS terminal 16091 16959 0.32 + 2 transcript_id "g1.t1"; gene_id "g1";

NKPW01072562.1_NKPW01009466.1 AUGUSTUS CDS 770 998 1 + 0 transcript_id "g1.t1"; gene_id "g1";

NKPW01072562.1_NKPW01009466.1 AUGUSTUS CDS 16091 16959 0.32 + 2 transcript_id "g1.t1"; gene_id "g1";

NKPW01072562.1_NKPW01009466.1 AUGUSTUS stop_codon 16957 16959 . + 0 transcript_id "g1.t1"; gene_id "g1";

coding sequence =
[atggctgggggccgtggggcgcgccgggaggggcctccaagggcaacggcagcgcgctgctcaatgcctcgcagcaggcgcctggcag
cggggagggcgcgccgtcgcgcccctcttggctggtcaccacgctcgccctcatcctcatcttcaccatcgtggtggacatcctgggcaacctcct
ggtcatcctgtccgtgtatcggaacaagaagctgaggaacgcaggaaatatatttgtggtgagcctagcagttgcagacctgctggtggccgtgta
tccataccccttggccctgacatctatcttcaacaatgggtggagcctgggctacctgcactgccagattagtggcttcctgatgggcttgagtgtcat
tggctccgtattcaatatcacggggattgccatcaaccgctattgctacatctgccacagtctccagtacgacaggttgtacagcaacaagaattccc
tctgctatgtgttcctgatatggatgttgacacttgtggcaatcgtgcccaacttgtgtattggaaccctgcggtacgacccgaggatctattcctgtac
attcacacagtccatcagttccgcgtacacgatagccgtggtggttttccatttcatggttcctatggtcatagtaatcttctgttacctaagaatctgggt
cctggttcttcaggtcagatggagggtgaaacctgacaacaaacccaaactgaaaccacaggacttcaggaattttgtcaccatgtttgtggtttttgt
actttttgccatttgctgggcgcctctaaacttcattggtcttgctgtggcctcagaccccgccagcatggtatccaggatcccagagtggctgtttgtg
gccagttactatatggcgtatttcaacagctgcctcaacgcaattatatatggactactgaaccaaaatttcaggcaggaatacaggagaattatagtc
tcactgtgcacagccaagatgttctttgtggatagctctaacaatgtcgcagatagaatcaaatgcaaaccctctccattaataaccaaccataaccta
ataaaggtggactccgtttaa]

protein sequence =
[MAGGPWGAPGGASKGNGSALLNASQQAPGSGEGAPSRPSWLVTTLALILIFTIVVDILGNLL
VILSVYRNKKLRNAGNIFVVSLAVADLLVAVYPYPLALTSIFNNGWSLGYLHCQISGFLMGL
SVIGSVFNITGIAINRYCYICHSLQYDRLYSNKNSLCYVFLIWMLTLVAIVPNLCIGTLRYDPRI
YSCTFTQSISSAYTIAVVVFHFMVPMVIVIFCYLRIWVLVLQVRWRVKPDNKPKLKPQDFRN
FVTMFVVFVLFAICWAPLNFIGLAVASDPASMVSRIPEWLFVASYYMAYFNSCLNAIIYGLL
NQNFRQEYRRIIVSLCTAKMFFVDSSNNVADRIKCKPSPLITNHNLIKVDSV]

Mtnr1b gene of Hippopotamus amphibius annotated by Augustus Software.

----- prediction on sequence number 1 (length = 40943, name = NKPW01013551.1) -----

#

Constraints/Hints:

(none)

Predicted genes for sequence number 1 on both strands

start gene g1

NKPW01013551.1 AUGUSTUS gene 25975 40822 1 - . g1

NKPW01013551.1 AUGUSTUS transcript 25975 40822 1 - . g1.t1

NKPW01013551.1 AUGUSTUS stop_codon 25975 25977 . - 0 transcript_id "g1.t1"; gene_id "g1";

NKPW01013551.1 AUGUSTUS terminal 25975 26813 1 - 2 transcript_id "g1.t1"; gene_id "g1";

NKPW01013551.1 AUGUSTUS initial 40600 40822 1 - 0 transcript_id "g1.t1"; gene_id "g1";

NKPW01013551.1 AUGUSTUS CDS 25975 26813 1 - 2 transcript_id "g1.t1"; gene_id "g1";

NKPW01013551.1 AUGUSTUS CDS 40600 40822 1 - 0 transcript_id "g1.t1"; gene_id "g1";

NKPW01013551.1 AUGUSTUS start_codon 40820 40822 . - 0 transcript_id "g1.t1"; gene_id "g1";

coding sequence =
[atgcccgagaaccgctccttcgccaactgctgcgaggcgggcggccgagccgagagcccaggctggactggggcgggcggcgcgcggcc
ctccgggacccccggcctccctgggtggcgcccgcgctgtccgccgtgctcatcgtcaccaccgcggtggacatcgtgggcaacctcctggtc
atcctctcggtgctcgggaaccgcaagctccggaacgcaggtaatttgttcttggtgagtctggcattggctgacctggcggtggccttgtacccct
acccgctaatcctcgtcgccatcttccacaacggctgggccctgggggaggcgcactgcaaggccagcgccttcgtgatgggtctgagcgtcat
cggctctgtcttcaacatcaccgccatcgccattaaccgctactgctacgtctgccgcagcgtgacctaccaccgcatctaccgccactggcacac
cgccctgtacatctgccttgtctggctgctcaccctgctggccgtggtacccaacttcttcttggggtccctggagtatgaccctcgcatctactcttgc
accttcatccagacggccagcgccgggtacacggtggccgtggtggtggtccacttcctcctccccatggctatcgtgtgctcctgttacctgcgca
tctgggtgctggtgctccgggcccgcaggaaggtcaaggcggagagcaagccgtgcctgtggtccaggaacgtccggagcttcctgaccatgtt
cgtggtgtttgtgatcttcgccatctgctgggcgcccctgaactgcattggcctcgctgtggccatcgacccagaagaagtggctccccgggtccc
agaggggctctttgtcacgagctacttcctggcttatttcaacagctgtcttaatgccatcatctacgggcttctgaaccagaacttccgcagggaata
caagaagatcgtctctgccctctggaacccacggcgctgccagcaggactcttccaagggcagccaggcggaggggccagagacccaagctc
cccgccgtggttaa]

protein sequence =
[MPENRSFANCCEAGGRAESPGWTGAGGARPSGTPRPPWVAPALSAVLIVTTAVDIVGNLLV
ILSVLGNRKLRNAGNLFLVSLALADLAVALYPYPLILVAIFHNGWALGEAHCKASAFVMGL
SVIGSVFNITAIAINRYCYVCRSVTYHRIYRHWHTALYICLVWLLTLLAVVPNFFLGSLEYDP
RIYSCTFIQTASAGYTVAVVVVHFLLPMAIVCSCYLRIWVLVLRARRKVKAESKPCLWSRNV

RSFLTMFVVFVIFAICWAPLNCIGLAVAIDPEEVAPRVPEGLFVTSYFLAYFNSCLNAIIYGLL
NQNFRREYKKIVSALWNPRRCQQDSSKGSQAEGPETQAPRRG]
